# A meta-analysis of the impact of TOE adoption on smart agriculture SMEs performance

**DOI:** 10.1371/journal.pone.0310105

**Published:** 2025-02-03

**Authors:** Adrian Nagy, Johan Tumiwa, Fitty Arie, Erdey László, Anas Ratib Alsoud, Main Al-Dalahmeh

**Affiliations:** 1 Institute of Economic Sciences, University of Debrecen, Debrecen, Hungary; 2 Department of Management, Sam Ratulangi University, Manado, North Celebes, Indonesia; 3 Institute of Economics and World Economy, University of Debrecen, Debrecen, Hungary; 4 Department of Business Technology, Al-Ahliyya Amman University, Amman, Jordan; 5 Hourani Center for Applied Scientific Research, Al-Ahliyya Amman University, Amman, Jordan; University of Southampton, MALAYSIA

## Abstract

**Background:**

Agricultural SMEs face distinct challenges due to factors such as weather, climate change, and commodity price changes. Technology has become essential in helping SMEs overcome these challenges and grow their businesses. The relationship between technology and SMEs in the agriculture sector covers various aspects, such as using hardware and software, digital applications, sensors, and e-commerce strategies to be examined in further depth through literature study.

**Problem statement:**

The implementation of the TOE (technology, organization, and environment) framework in smart agriculture faces several challenges. To overcome these challenges, an integrated approach is needed that involves technological capacity building, organizational management changes, and adequate policy and infrastructure support to help SMEs in the agricultural sector develop their businesses.

**Objectives:**

This research aims to demonstrate and identify how TOE plays an important role in the performance of SMEs, particularly with regard to agriculture in order to improve agricultural productivity, efficiency, and sustainability while enabling access to broader markets in several countries. This study employs a meta-analysis method using a quantitative approach taken by each publication, which typically used SEM.

**Methods:**

PRISMA technique was used to examine evidence from clinical trials, and clinical significance was determined using the GRADE approach. Statistical analysis was performed using the Fisher test to combine the results of several studies and Cohen’s approach to interpreting effect sizes.

**Findings:**

The results of this study are in line with the findings of 27 previous studies which showed a direct positive relationship between TOE construction and the performance of agricultural SMEs, with variables including technological factors, organizational factors, environmental factors, and SME performance. The synergy between technology adoption by agricultural SMEs and Industry 4.0 can increase connectivity and automation in the agricultural sector. However, it is important to remember that adopting TOE to realize the smart agriculture concept has its own challenges and risks, such as resource management (technology), good organizational management (organization), and internal and external organizational environments (environments), including intense competition

**Research implication:**

TOE adoption improves access to information about competitors and customers, providing practitioners and decision-makers with a clearer understanding. It enables focus on factors with a significant impact on TOE adoption, so that they are more independent in developing effective business concepts that are adaptive to the era of agricultural technology 4.0.

## Introduction

Agriculture sector should adopt new technologies to progress in the technological and advanced world. SMEs play an essential role in the global economy, and the agricultural sector is no exception. They are often the backbone of local farm production, contributing to food security and economic development. Growth in the agricultural sector is necessary to develop the country’s economic conditions and provide extensive employment opportunities for the community [1]. Unfortunately, many farmers continue to adopt old farming methods, which results in low yields.

This research aims to harness the power of meta-analysis to unveil the multifaceted impacts of the Technology-Organization-Environment (TOE) framework on the performance of Small and Medium Enterprises (SMEs) in the agriculture sector. By synthesizing data across diverse studies, the objective is to provide a comprehensive evaluation of how technological advancements, organizational strategies, and environmental considerations collectively enhance agricultural productivity, efficiency, and sustainability. The research seeks to identify key barriers and facilitators to the adoption of smart agriculture practices, offering insights into the role of technological innovation in navigating the challenges of climate change and market dynamics. Ultimately, this study aspires to deliver evidence-based recommendations that can guide policymakers, technology developers, and agricultural practitioners toward fostering an enabling environment for the effective implementation of the TOE framework, thus paving the way for a more resilient and sustainable agricultural future.

**Fig 1:**
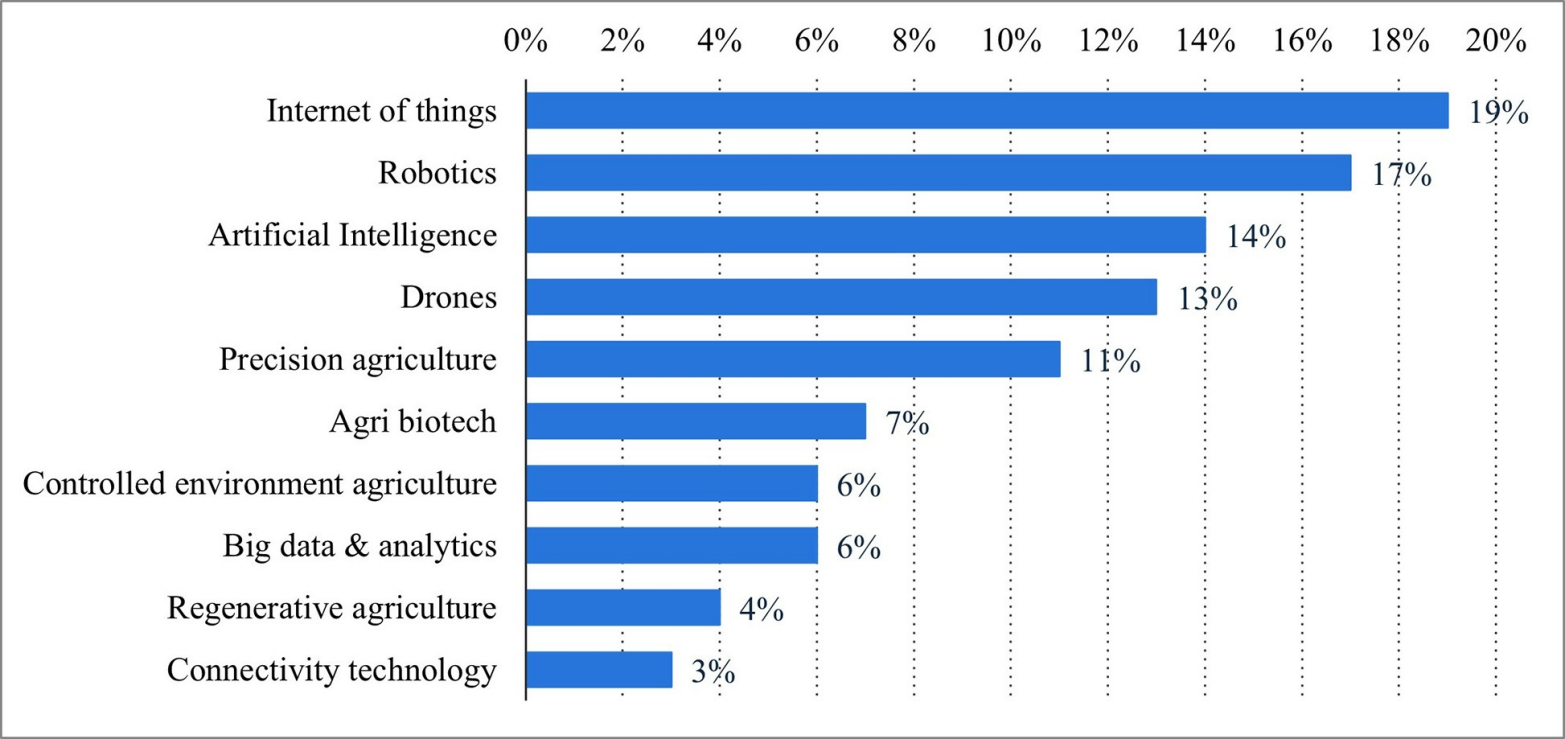
Bar chart 1. Global share of leading agricultural technology advancements in 2022. Source: Statista 2023.

Issues regarding agriculture have always hampered the development of the country. By upgrading traditional farming methods, smart farming is the only solution to this challenge [2]. Hence, this project aims to make agriculture bright using automation and IoT technology. Internet of Things (IoT) is a concept where various physical devices such as sensors, machines, vehicles, and household appliances are connected to the internet and are able to collect, play, and process data and control more efficiently. This technology is used in various fields, including agriculture to increase productivity, efficiency, and comfort. The rapid information and communication technology era has changed many aspects of human life, including the agricultural sector. Technologies such as IoT, sensors, drones, AI, and data analytics have opened up new opportunities in agricultural management. All sectors, including agriculture, are forced to adapt and use internet based digital technologies in order to develop smart farming specifically for the SME sector as a response to COVID pandemic 19 and industrial revolution 4.0 [3]. Smart agriculture promises higher efficiency in resource use, and increased productivity, and promotes environmental sustainability. It is a relevant solution in the face of challenges such as a growing world population and climate change [4]. Agricultural development is based on increasing productivity and the limitations of the times, and advances in science and technology are driving the agricultural revolution [5]. By a factor of 19, the Internet of Things (IoT) will be the most impactful agricultural technology advancement in 2022. IoT is a sensor that can monitor crops in real time and provide farmers with information into crops that were previously obtained manually. With presentations of 17% and 14%, robotics and artificial intelligence are the second and third most influential AG technology innovations in 2022. See Fig 1.

Although technology has enormous potential for the sustainability of SMEs in agriculture, it is not easy for SMEs to apply existing technology on a massive scale, requiring synergy for management in managing efficient and effective information systems to be adopted into technology. The challenges include limited access to capital, technological knowledge, infrastructure, and government support. These difficulties encourage the agricultural industry to make the shift to smart agriculture by deploying the Internet of Things (IoT) and big data technologies in order to improve operational effectiveness and productivity [6]. Existing advanced solutions and technologies are being integrated into IoT including wireless sensor networks, cognitive radio-based networks, ad hoc networks, cloud computing, big data, and end-user applications.

The emphasis on IoT in Agriculture SMEs is contained explicitly in the TOE concept, a framework used to analyze how technology affects organizations and their environment; IoT is one example of technology that can be analyzed through the TOE framework [7]. According to Kruger & Steyn [8] there are several benefits of using technology such as IoT, sensors, data analytics, and others can improve agricultural productivity and efficiency, farming organizations can develop better strategies to adopt relevant technologies, TOE helps in understanding how technology adoption can impact agricultural organizations internally, identifying associated opportunities or threats, help improve the quality of agricultural products, improve modern and sustainable agriculture. Understanding and applying the TOE framework can help the agricultural sector to better adapt to technological and environmental changes while increasing yields and overall sustainability [9]. The main problems that users experience in technology adoption include budget, limited knowledge, privacy, and security issues. These issues also cause delays in technology adoption. Many sectors have discovered that technological infrastructure is a crucial aspect in assuring the managerial success of new technologies. The most difficult aspect of applying technology in agriculture is ensuring that the model maintains its performance under all environmental conditions, such as rainfall, humidity, sunlight, temperature, and water availability [10].

In contrast to the research conducted by Shilomboleni [11] which systematically analyzes the concepts and contexts that frame the smart agriculture discourse through the academic literature, indicating that research on TOE needs to be carried out not only focusing on smart agriculture but also a scientific approach and the context of geographical context. If TOE is to be applied to farmers around the world, then interdisciplinary research underpinned by broad socio-economic and political contexts is essential to understand how these differences can be used. Based on the background above, it can be concluded that TOE is a technology system to support production effectiveness and efficiency in SMEs in the agriculture sector. To describe the adoption of TOE for agricultural performance SMEs globally, a meta-analysis study is needed. Several previous studies have investigated technology adoption for agriculture and SMEs. However, there is still room to integrate this understanding into the context of smart agriculture transformation for SMEs in a more comprehensive manner. Therefore, this research further examines using the concept of Meta-Analysis on how the adoption of smart agriculture affects SMEs in the agricultural sector and how various challenges can be overcome. With a better understanding of this topic, we can identify the necessary measures to support the growth and sustainability of SMEs in the technological era in the future and for future research. This study helps identify which technologies are most relevant and beneficial for smart farming SMEs by understanding IoT platforms, SMEs can more easily select and implement technologies that suit their needs and provide insights into how SMEs can prepare themselves organizationally to adopt technologies to move to smart farming. This study also highlights the importance of the external environment, including government policies, regulations, and support from third parties such as technology providers and business partners.

## Literature review

### Smart agriculture concept

According to Patel et al. [12] smart farming is an approach to agriculture that is managed using software and monitored by sensors integrated with advanced technology for informed and data-driven decision making. Smart agriculture, also referred to as precision agriculture or smart farming, is an agricultural management concept that leverages advanced technologies to optimize farming operations [13]. Using advanced technology, smart agriculture aims to address the challenges of modern agriculture, including a growing population, climate change, and increased demand for agricultural products. Technological development in agriculture goes through several stages and processes. Agriculture 1.0 was defined by animal power; Agriculture 2.0 was defined by the combustion engine; and Agriculture 3.0 has recently been defined by guiding systems and precision farming. Agriculture 4.0 agriculture activities are currently linked to cloud computing [14]. Smart agriculture through the use of information and communication technology to support agricultural businesses that are more efficient, productive and profitable [15]. The benefits of smart agriculture include increased crop yields, reduced resource wastage, improved resource management, enhanced pest and disease control, and optimized use of fertilizers and irrigation. Innovative approaches in technology promote the stabilization, development, and competitiveness of small and medium-sized enterprises (SMEs), especially in rural areas where agriculture is the backbone of the economy [16]. Despite the numerous benefits, smart agriculture also faces challenges and constraints. One of the related issues was discussed by Santiteerakul et al. [17] who stated that smart agriculture has the potential to hamper sustainable development because the lack of resource utilization and energy use, as well as pollution due to toxic chemicals, cannot continue at the same level as today. While there is potential to reduce environmental impacts, for example through more efficient water use, the implementation of technologies can also lead to unintended environmental impacts if not managed properly. The fundamental difficulty that proponents of the notion of smart agriculture have is how to establish a framework for the term’s development so that it is connected with sustainability [18]. This difficulty underscores the necessity of adopting a comprehensive approach that carefully considers and addresses the multifaceted impacts of smart agriculture technologies. When adopting smart agriculture technology, it is essential to consider these various aspects and find ways to minimize negative impacts while maximizing benefits for farmers, society, and the environment [19]. Through this study, the adoption of the TOE framework in Smart agriculture SMEs in understanding the critical factors of the importance of utilizing technology wisely and relevantly includes information technology, sensors, digital applications, and other modern agricultural tools supported by IoT.

### Internet of Things (IoT) in agriculture

IoT is important in developing smart agriculture. Based on Dwiv et al. [20] IoT enables increased internet-connected monitoring and censoring, allows automated control of various agricultural devices and systems, integrates data from multiple sources, such as sensors, devices, and farm management systems, provides valuable insights into agricultural conditions, and ensures that farmers have access to agricultural data in real-time, which can help them take immediate action if there are problems or changes in situations. Furthermore, the research conducted by Antony et al. [21] states that the expansion of small-scale precision devices connected to the Internet supported by the IoT has the potential to increase opportunities for agricultural food security efforts and accelerate the journey of low and middle income countries toward self-reliance, as IoT sensors are able to provide information about agriculture and then act on user input [22]. The proliferation of small-scale precision devices integrated with the Internet, facilitated by IoT, holds substantial potential for enhancing agricultural food security and promoting self-sufficiency in low and middle-income countries. These IoT sensors, capable of delivering real-time agricultural data and executing actions based on user inputs, have the capacity to transform farming methodologies. Nonetheless, the effective deployment of these technologies necessitates addressing critical issues related to infrastructure preparedness, data management, and the training of users in these regions. In order to achieve the goal of more efficient, sustainable, and productive agriculture, IoT has become an integral part of smart agriculture. In agriculture, IoT has been widely used in management systems, monitoring systems, control systems, and unmanned machines in various forms of wireless communication technologies used in agriculture, such as Wi-Fi, remote wide area networks (LoRaWAN), cellular communication (2G, 3G, and 4G), ZigBee, and Bluetooth [23]. With the adoption of these technologies, farmers can optimise their operations, reduce risks, and increase their yields. However, the issue of IoT is gaining importance and experiencing dramatic development in the agriculture sector and brings many new technological innovations and new problems [24]. However, it also brings new challenges related to data security, technology skill requirements, implementation costs, and dependency on technology. Therefore, a balanced approach is needed to maximize the benefits while mitigating the risks that arise through this research.

### IoT (Internet of Things) and TOE (Technology-Organization-Environment)

The relationship between IoT and Technology-Organization-Environment (TOE) is a concept that helps explain how IoT technologies interact with organizational and environmental factors to influence the adoption and implementation of IoT in a business or organizational context [25]. The implementation of TOE has been widely studied and is a productive area of organizational-level inquiry [26]. The research on IoT adoption within organizations, especially SMEs, is still emerging. To date, most studies have concentrated on applying established frameworks like Diffusion of Innovation (DOI) and Technology-Organization-Environment (TOE) to understand the factors influencing IoT adoption [27]. However, this focus on traditional models has not sufficiently addressed the rapidly evolving nature of IoT technology and its integration into diverse organizational contexts. Consequently, rather than fostering a unified approach to IoT adoption that encourages collaboration and standardization, the current trajectory of research has led to a fragmented IoT domain characterized by a lack of cohesion and widespread adoption strategies [28]. Therefore, we use the TOE framework as the theoretical basis because it includes essential components that DOI theory does not [7]. Furthermore, IoT controls how technology is applied and can influence organizational strategies and decisions [29], synergy between IoT and TOE underscores the importance of understanding that technology adoption is not just about implementing hardware and software but involves changes in organizational culture, setting the right policies, and managing the external environment [30]. The interaction between IoT and the Technology-Organization-Environment (TOE) framework highlights that effective technology adoption goes beyond direct hardware and software implementation. This holistic approach underscores the complex nature of technology integration, which requires strategic alignment across multiple organizational dimensions to harness its full potential. Overall, the TOE concept helps organizations understand the dynamics of IoT adoption and implementation in a technology-organization-environment context, enabling them to make better decisions and maximize the benefits of the technology [25]. Through research can be used by policy makers to develop policies that support technology adoption in the agricultural sector, such as tax incentives, subsidies, and training programs so that they can develop more effective business strategies based on a better understanding of the factors that influence technology adoption. By adopting smart technology, agricultural SMEs can increase their competitiveness in local and international markets and continue to innovate and adapt to changing markets and environments efficiently and effectively without having to ignore environmental sustainability by optimizing the use of resources such as water, fertilizers, and labor, which ultimately increases operational efficiency and reduces operational costs through process automation, waste reduction, and increased productivity. Formally, we postulate:

**H.1** To improve smart agriculture, TOE adoption has a positive and significant impact on (a) SMEs’ overall agricultural performance, (b) technological performance, (c) organizational performance, and (d) environmental performance.
**H.2** To improve smart agriculture, TOE adoption has a negative and minor impact on (a) SMEs’ overall farm performance, (b) technological performance, (c) organizational performance, and (d) environmental performance.

## Methodology

### Research design

This study uses quantitative methodologies in conjunction with a meta-analysis procedure. Meta-analysis (MA) is a process for summarizing the findings of previous research by calculating statistical correlations between study values and explanatory variables that incorporate heterogeneity within and between studies [31]. The main purpose of a meta-analysis is to provide a stronger and more general summary of the topic than a single study can provide [32]. The research involved searching for relevant literature reviews between 2019–2023 in order to compile a systematic review. However, as part of the methodological process, the searches had time constraints that required no searches to be conducted more than one year from submission, to ensure that the information included in the systematic review remained relevant and up-to-date the searches were conducted using various relevant databases and keywords appropriate to the research topic from databases, such as Web of Science, Scopus, and Google Scholar. As a result, we began the review with a bibliographic keyword search to select articles that investigated the relationship between TOE adoption and SME performance in the agriculture sector from 2020 to 2023 in internationally recognized management databases, such as Web of Science, Scopus, and Google Scholar. The searches were carried out by using the Boolean operation to enter the following keywords in the title, abstract and keyword fields of the database boolean operations TITLE-ABS-KEY *(toe) OR (iot) AND (smart AND agriculture) AND (sme) AND (sem) AND (LIMIT-TO (SUBJAREA*, *"BUSI")) AND (LIMIT-TO (DOCTYPE*, *"ar")) AND (LIMIT-TO (LANGUAGE*, *"English"))*.This research focused on the data and impact of TOE adoption on the development of smart agriculture SMEs in different countries.

In this meta-analysis, handling missing data was a critical step to ensure the integrity and reliability of the findings. To minimize the occurrence of missing data, the studies included were carefully selected based on stringent inclusion and exclusion criteria. However, in instances where data were incomplete, we employed multiple imputation techniques, which are consistent with best practices in meta-analytic research.

Multiple imputation involves creating several imputed datasets to estimate missing values, thereby preserving the statistical power of the analysis and preventing potential bias. The process begins by identifying the variables with missing data and assessing the missing data mechanism—whether it is Missing Completely at Random (MCAR), Missing at Random (MAR), or Missing Not at Random (MNAR). This assessment informs the choice of imputation method.

For this analysis, we used Predictive Mean Matching (PMM) to impute the missing values across multiple datasets. Each imputed dataset was analyzed independently to ensure that the variability introduced by imputation was accurately reflected. The results from these analyses were then pooled to provide comprehensive estimates, which consider both within- and between-imputation variability. This approach allowed us to maintain the robustness of the statistical analysis and ensure that the final results were not biased by the missing data.

Furthermore, to assess the impact of the imputed data on the overall results, sensitivity analyses were conducted. These analyses confirmed that the conclusions drawn from the imputed datasets were consistent with those obtained from the original data, indicating that the imputation did not introduce any significant bias.

The detailed process and results of the multiple imputation are provided in the supplementary information, which includes the specific techniques used, as well as the raw and processed datasets. This ensures transparency and allows for replication and further exploration of our findings, in line with the journal’s requirements.

### Eligibility criteria

**Fig 2:**
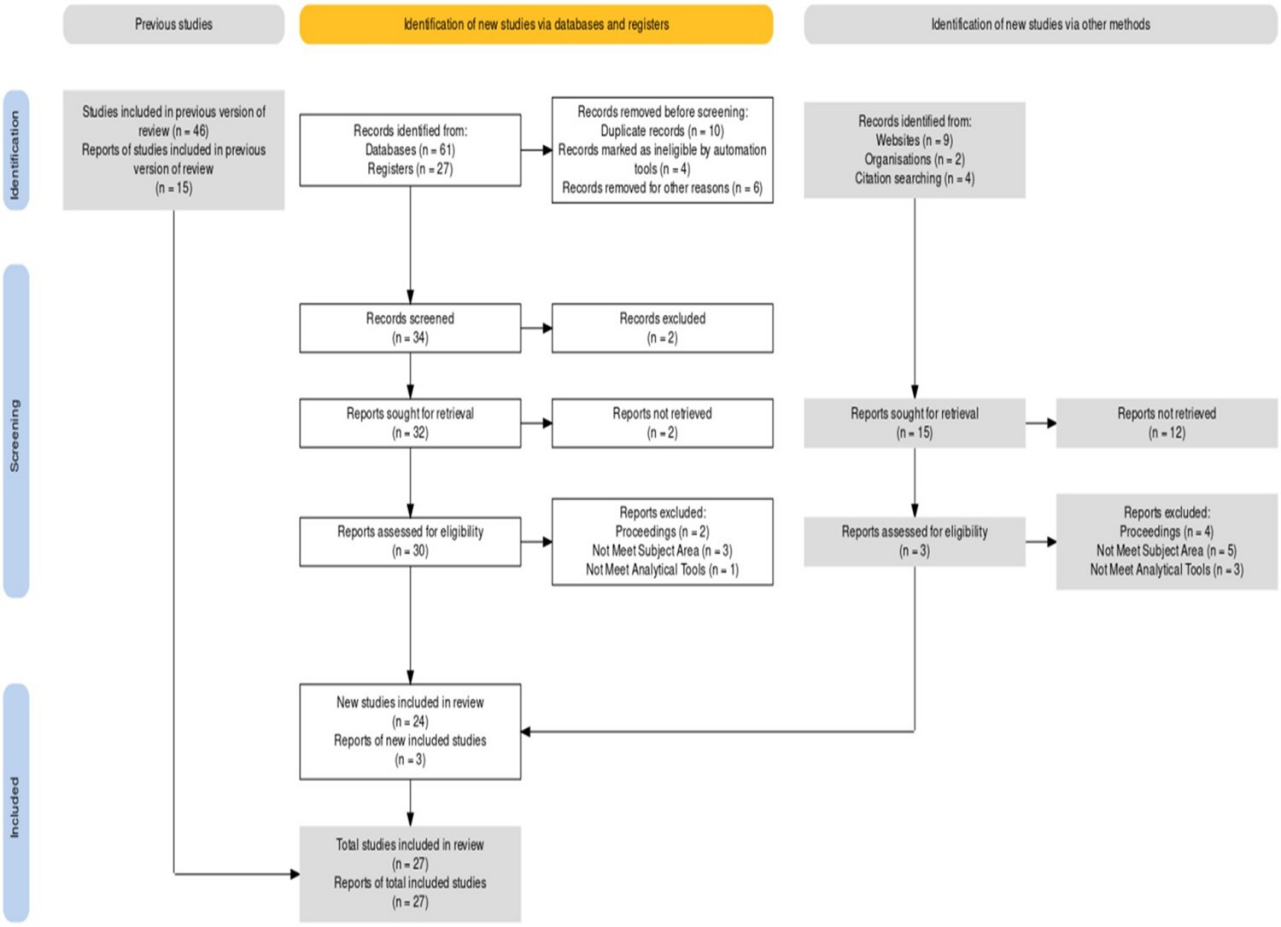
PRISMA flowchart for systematic reviews. Source: Author’s data processing, 2024.

The reviewers create qualifying criteria before beginning the process of searching, discovering, and retrieving the research needed to address evidence-based practice challenges [33]. After an initial screening of selected articles by reading abstracts and identifying inclusion and exclusion criteria, SLR will usually report the search funnel and the authors’ inclusion and exclusion criteria applied to narrow down a search as a show by Fig 2. The emphasis at this stage of the selection process is on excluding studies that clearly meet the exclusion criteria. Studies will be removed from a candidate study of a literature review if their title and abstract clearly disqualify them in a systematic manner that allows the researcher to draw valid and reliable conclusions about the effect of the intervention on the disorder under consideration. After the inclusion and exclusion stages, 27 (Scopus, WoS, and Google scholar) scientific articles were identified, with 61 observations from 2019 to 2023 that met the specific and inclusion criteria of our model. Relevant studies were then coded based on their conceptual features of TOE adoption and methodological characteristics and SMEs agriculture contextually. There were several articles that were not included (exclusion criteria) because they did not meet the objectives and qualifications of the field of science and analytical methods that will be used in this study.

### Data encoding

The next stage in this research method is coding to collect, group and organize data from various studies that will be included in the meta-analysis. Data coding is accomplished by coding the factors utilized to provide more concentrated information in estimating the influence of adoption on the performance of agricultural sector SMEs, as demonstrated by Table 1. This data can include results, sample size, effect statistics, information about the study population, research design, and others. In this study, data is coded by clearly describing the characteristics of the publications used or converted into a consistent format, such as publication year, country of origin, publication sample (N), correlation value (r, x, y), and a description containing journal accreditation/reputation information [34].

**Table 1:** Comparison data coding.

No	Author	Year	Country	r	N	Source
**1**	Qalati et al.	2021	Pakistan	0.559	423	Scopus
**2**	Setiyani and Yeny Rostiani	2021	Indonesia	0.304	301	WoS
**3**	Opasvitayarux et al.	2022	Thailand	0.642	197	Scopus
**4**	Andaregie and Astatkie	2022	Euthopia	0.53	327	WoS
**5**	Shahadat, et al.	2023	India	0.698	535	Scopus
**6**	Bawono et al.	2022	Indonesia	0.687	120	Google Scholar
**7**	Stjepić et al.	2021	Croatia	0.377	100	Scopus
**8**	Nguyen et al.	2023	Vietnam	0.548	234	Google Scholar
**9**	Trawnih et al.	2023	Jordan	0.423	218	Scopus
**10**	Sugandini et al.	2023	Indonesia	0.678	280	Google Scholar
**11**	Boakye et al.	2023	Ghana	0.452	214	WoS
**12**	Ramdani et al.	2020	Northwest of England	0.333	102	Scopus
**13**	Deng et al.	2022	China	0.286	330	Scopus
**14**	Atan et al.	2023	Malaysia	0.348	241	Scopus
**15**	Thaha et al.	2022	Indonesia	0.672	389	Scopus
**16**	Bag et al.	2023	South Africa	0.598	311	Scopus
**17**	Yoon et al.	2020	Korea	0.437	232	Scopus
**18**	Amini and Javid	2023	Malaysia	0.919	170	WoS
**19**	Qalati et al.	2022	Pakistan	0.738	381	Scopus
**20**	Giampietri and Trestini	2020	Italy	0.644	94	WoS
**21**	Liu and Cho	2022	China	0.516	373	Scopus
**22**	Lutfi et al.	2022	Jordan	0.793	184	Scopus
**23**	Ahmad and Siraj	2023	North India	0.825	384	Google Scholar
**24**	Abdullahi et al.	2021	Somalia	0.713	107	WoS
**25**	Junior et al.	2019	Brazil	0.38	375	Scopus
**26**	Awa et al.	2019	Nigeria	0.456	373	Scopus
**27**	Dadhich and Hiran	2022	India	0.963	395	Scopus

Source: Author’s data processing, 2024

The following is the descriptive analysis of the article that is used in this research:

Our research examines studies from 19 countries between 2019 and 2023, authored by a collective of 93 unique researchers, highlighting a burgeoning global interest in the performance of SMEs. This period, marked by significant global challenges, has evidently accelerated academic pursuits into SME dynamics, reflecting a concerted, interdisciplinary effort to understand their pivotal role across diverse economic and cultural landscapes.

**Fig 3:**
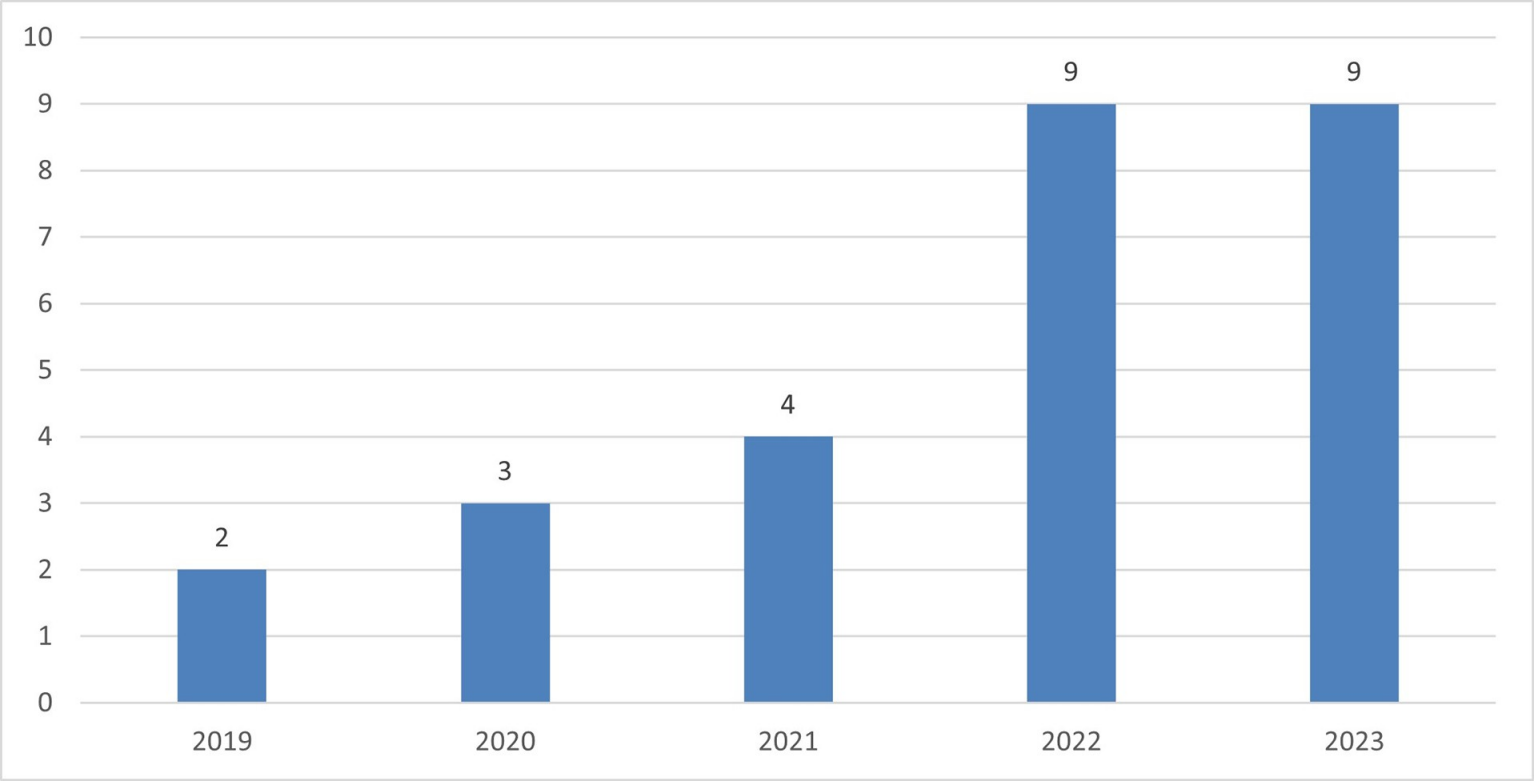
Publication frequency by year. Source: Author’s data processing, 2024.

We reveal a notable trend in the volume of research shown by the period of our systematic literature review on meta-analysis, spanning from 2020 to 2023. Fig 3 shows that our time frame starts from 2019 with 2 publications. In 2020, the publication increased to 3 studies, reflecting a growing interest in the field. The level of interest in TOE among SMEs, utilizing SEM and obtaining an R2 result has shown a slight decline in 2021, with only 4 studies conducted. In 2022, there has been a noteworthy rise in the number of studies carried out on this subject matter. Nine studies have indicated a peak in research activity, and the fact that the number of studies has remained at nine in 2023 might be a sign of stabilization in research output or a shift in focus within the academic community.

### Data analysis

Generating meta-analysis, study participants are organized into layers within studies, resulting in a multilevel data structure. The typical random effects model has a randomized study effect, but additional random effects can be established to account for various dependent impact sizes within or between studies [35]. Meanwhile, data analysis in this study was carried out through the following steps: (1) analysis of the characteristics of the research sample (show by Table 2). Sample characteristics in the context of meta-analysis refer to numerous aspects or parameters linked to the sample of persons or units evaluated in the studies included in the meta-analysis; in this investigation, 27 scientific papers were used, with an observed number of 1.016. (eligible).

**Table 2:** Characteristic of sample.

Test of Excess Significance | Significant Findings	
Observed Number of Significant Findings	27
Expected Number of Significant Findings	27
Observed Number / Expected Number	1.016

Source: Author’s data processing, 2024

(2) data coding (Table 1); (3) formula conversion of t and F values to r-correlation values (Table 3):

$$z=0.5\;x\;\ln\;\frac{1+r}{1-r}\;\ldots \ldots \ldots \ldots \ldots \;\mathrm{Effect}\;\mathrm{size}\;\mathrm{of}\;\mathrm{each}\;\mathrm{study}\;(a)$$

$$Vz=\frac{1}{n-3}\;\ldots \ldots \ldots \ldots \ldots .\mathrm{Varian}\;\mathrm{of}\;\mathrm{effect}\;\mathrm{size}\;(b)$$

$$SEz=\sqrt{V}z\;\ldots \ldots \ldots \ldots \ldots \ldots \mathrm{Standard}\;\mathrm{error}\;\mathrm{of}\;\mathrm{effect}\;\mathrm{size}\;(c)$$

(4) Effect size heterogeneity test (findings); (5) computing summary effect or mean effect size (findings); (6) generating forest plots and funnel plots (findings); (7) hypothesis testing (findings); and (8) screening for publication bias (findings).

**Table 3:** Conversion of r and N values to z,Vz and SEz values.

Study	r	N	z	Vz	SEz
Qalati et al.	0.559	423	0.631	0.002	0.049
Setiyani and Yeny Rostiani	0.304	301	0.314	0.003	0.058
Opasvitayarux et al.	0.642	197	0.762	0.005	0.072
Andaregie Adino and Astatkie Tessema	0.53	327	0.590	0.003	0.056
Shahadat, M et al.	0.698	535	0.863	0.002	0.043
Bawono et al.	0.687	120	0.842	0.009	0.092
Stjepić et al.	0.377	100	0.397	0.010	0.102
Nguyen Phuc Hien et al.	0.548	234	0.616	0.004	0.066
Trawnih et al.	0.423	218	0.451	0.005	0.068
Dyah Sugandini et al.	0.678	280	0.825	0.004	0.060
Boakye et al.	0.452	214	0.487	0.005	0.069
Ramdani et al.	0.333	102	0.346	0.010	0.101
Deng et al.	0.286	330	0.294	0.003	0.055
Atan et al.	0.348	241	0.363	0.004	0.065
Thaha et al.	0.672	389	0.814	0.003	0.051
Bag et al.	0.598	311	0.690	0.003	0.057
Yoon et al.	0.437	232	0.469	0.004	0.066
Amini and Javid	0.919	170	1.583	0.006	0.077
Qalati et al.	0.738	381	0.946	0.003	0.051
Giampietri and Trestini	0.644	94	0.765	0.011	0.105
Liu and Cho	0.516	373	0.571	0.003	0.052
Lutfi et al.	0.793	184	1.079	0.006	0.074
Ahmad and Siraj	0.825	384	1.172	0.003	0.051
Abdullahi et al.	0.713	107	0.893	0.010	0.098
Junior et al.	0.38	375	0.400	0.003	0.052
Awa et al.	0.456	373	0.492	0.003	0.052
Dadhich and Hiran	0.963	395	1.986	0.003	0.051

Source: Author’s data processing, 2024

Cohen’s effect size was employed in our analysis to quantify the magnitude of the effects observed across studies, offering a standardized measure that facilitates comparison between different studies and outcomes. This metric is particularly valuable because it allows us to interpret the practical significance of results, beyond merely determining statistical significance. However, we recognize that effect size alone cannot address potential biases within the studies. Therefore, we have meticulously assessed and addressed the risk of bias in each article used to extract data. This was done by providing detailed responses to each of the signaling questions across all relevant domains, ensuring that the effect sizes calculated are not only robust but also reflect the true magnitude of the effects, free from distortions caused by bias. Additionally, we have included a comprehensive table detailing the bias assessment for each article in the supplementary file, which supports our rigorous approach to ensuring the reliability of our findings. The following Table 3 provides the extracted data from the selected articles and then further calculated for the z,Vz and SEz values.

The software used in this study is Jamovi 2.3 and for the effect size criteria using Cohen’s criteria presented in Table 4 below.

**Table 4:** Cohen’s effect magnitude threshold.

Criteria	Value
Weak Effect	< 0+/-.1
Modest effect	< 0+/-.3
Moderate effect	< 0+/-.5
Strong effect	< 0+/-.8
Very strong effect	> 0+/-.68

Source: Author’s data processing, 2024

Based on the results of the conversion analysis of r and N values (Table 3), each article or sample used in this study has the smallest effect size (z) value of 0.304 and the largest is 0.963. Based on Cohen’s assumption, shown in the Table 4, the effect size for this study is at a modest-very strong effect, in other words, it is feasible to be tested as a whole in the discussion section.

## Findings

Regarding to the 27 research publication with specific criteria of analysis various values of N, z, Vz and SEz were obtained for each study. After the Z values were converted to R values, they were tested for heterogeneity. Heterogeneity in meta-analysis refers to the variation or differences between the results of the studies collected for synthesis. This is an important concept in meta-analysis as it can affect the final conclusion of the analysis. Meanwhile, the results of the heterogeneity test are shown in Table 5.

**Table 5:** Heterogeneity test.

Statistics on Heterogeneous Populations							
Tau	Tau^2^	I^2^	H^2^	R^2^	df	Q	p
0.385	0.1481 (SE = 0.0424)	97.55%	40.810	.	26.000	1171.391	< .001

Source: Author’s data processing, 2024

Table 5 shows the most common statistical test for heterogeneity in meta-analysis which is the Q test. The Q test compares the observed variation between study results with the expected variation due to sampling error. In this study, the Q test produced a p-value of <0.01, indicating significant heterogeneity (<0.05 or 0.1). The Fisher r-to-z transformed correlation coefficient was used as the outcome metric in the investigation. The data was fitted with a random-effects model. The constrained maximum-likelihood estimator was used to estimate the level of heterogeneity (i.e., tau^2^) [36]. The tau-square statistic reflects the absolute heterogeneity and can be used to calculate confidence and prediction intervals for meta-analysis results. Parameter *τ* is the variance of the effect size. In fact (true effect size), a good value of *T* is If *T*^2^ > 0 or *T* > 0, in this study, the value of *T*^2^ is 0.1481> 0. Moreover, apart from using parameters *Q* and *τ*^2^. Heterogeneity tests can also be carried out using the parameter *I*^2^. The parameter *I*^2^ is the ratio of true heterogeneity to the total variance of the observed effect. A good value of *I* is close to 100%. The *I*^2^ value, which is getting closer to 100%, indicates that the effect size between studies is increasingly heterogeneous; Table 5 shows the *I*^2^ value of 97.55% in this study. In addition, the heterogeneous data indicates that there may be potential to investigate other moderator variables that affect the relationship between TOE and SMEs Agriculture performance.

The rank correlation test and the regression test, which use the standard error of the observed results as predictors, are shown in Table 6 to examine funnel plot asymmetry. A total of k = 27 studies were included in the analysis. Fisher r-to-z transformed correlation coefficients discovered varied from 0.2942 to 1.9857, with the bulk of estimations (100%) being positive. Based on the random-effects model, the estimated average Fisher r-to-z transformed correlation coefficient was \hat{\mu} = 0.7280 (95% CI: 0.5806 to 0.8755). As a result, the average result deviated significantly from zero (z = 9.6785, p 0.0001). The genuine outcomes appear to be heterogeneous, according to the Q-test. Q (26) = 1171.3911, p 0.0001, tau2 = 0.1481, and I2 = 97.5496%. -0.0405 to 1.4966 is the 95% prediction interval for the true outcomes. As a result, while the average outcome is projected to be favorable, the true outcome in some studies may be negative. An examination of the residuals revealed that one study by [26] had a value greater than 3.1130 and might be considered an outlier in the context of this model. According to Cook’s distances, could be deemed overly influential. The rank correlation and regression tests both showed no funnel plot asymmetry (p = 0.9004 and p = 0.6704, respectively). The findings of the analysis with random effects reveal a p-value of 0.01 indicating that the influence of TOE adoption on SMEs Smart Agriculture performance is significant. Meanwhile, the calculated standard error value of 0.728 [0.581; 0.875] indicates the extent of the effect of TOE on SMEs Smart Agriculture performance. Based on Cohen’s effect size criteria, the projected value of the standard error can be classified as having a large influence. Furthermore, the outcomes of the meta-analysis study can be summarized graphically using the Forest Plot.

**Table 6:** Summary effect or mean effect size.

Model of random effects (k = 27)						
	Estimate	se	Z	p	CI Lower Bound	CI Upper Bound
**Intercept**	0.728	0.0752	9.68	< .001	0.581	0.875
	.	.	.	.	.	.
**Tau2 Estimator: Maximum-Likelihood Restricted**						

Source: Author’s data processing, 2024

**Fig 4:**
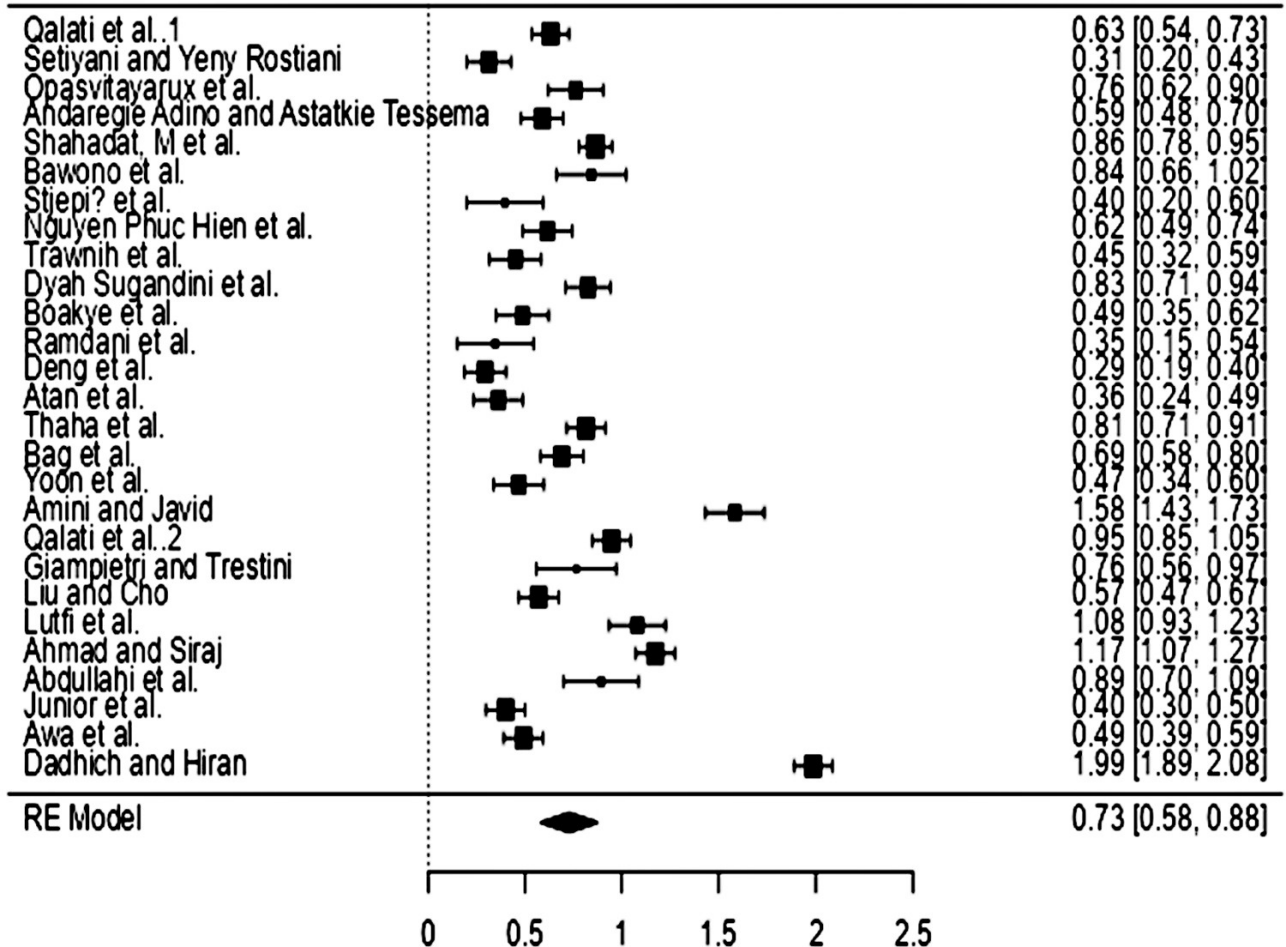
Forest plot. Source: Author’s data processing, 2024.

Forest plot is a visual tool used in meta-analysis to illustrate the results of synthesized studies. Forest plots help readers to see the estimated effect of each study as well as the estimated pooled effect of all the studies that have been analyzed [37]. The center of the box represents the point estimate of the effect (effect size; such as risk ratio, odds ratio, or mean difference), whose size (area) is proportional to the study weight. The diamond shape represents the total pooled effect of the included studies. The width of the diamond represents the confidence interval for the overall effect. The further to the right, the larger the effect size value. The bigger the plot, the more important or very important it is. In addition, the RE model displays the pooled effect size values of the analyzed studies in diamond plots. Horizontal lines extending from each study show confidence intervals for each study’s effect size. Each forest plot has a vertical line called the "no effect" line, which corresponds to a value of 1 for binary outcomes like risk ratios or odds ratios and a value of 0 for continuous outcomes. The difference in outcome between the intervention and the comparator is not significant if the 95% CI of the individual study or pooled estimate passes the zero effect line. In this study, the value of the RE model corresponds to the standard error estimate, which is 0.73 as a shown by Fig 4. It is possible to conclude that the forest plot represents a summary of the analysis.

**Fig 5:**
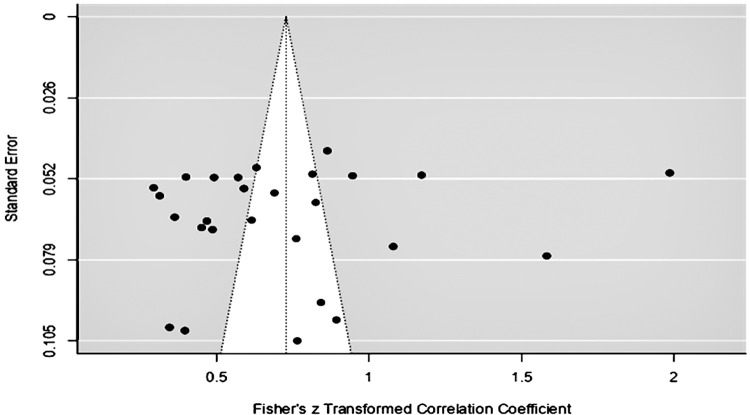
Funnel plot. Source: Author’s data processing, 2024.

A funnel plot is a simple scatter plot of estimated treatment effects based on individual studies compared with a measure of study size. The term "funnel plot" refers to the precision with which the estimate of the possible treatment impact rises as the sample size of the component research grows [38]. A solid meta-analytic study avoids publication bias in its analysis. Data analysis employing the funnel plot, Egger test, and Fail-Safe N technique were used to assess publication bias. The value obtained by dividing the overall impact size by the value represented by the line drawn (Fig 5) is the value of the summary effect size. If the distribution of effect size values on the right and left sides of the hemisphere is the same, the plot is said to be symmetrical. A funnel plot is shown by Fig 5 from this meta-analysis study. A diagonal line representing the 95% confidence limit for the overall treatment impact, H, can be added to the funnel plot to make it easier to read. For each standard error on the vertical axis, compute [Summary impact estimate—(1.96 standard error)] and [Summary effect estimate + (1.96 standard error)]. These depict the predicted distribution of studies in the absence of heterogeneity or selection bias: if no heterogeneity existed, 95% of the research should fall within the funnel represented by these lines. Because these lines do not represent strict 95% confidence levels, they are referred to as "pseudo-95%" confidence limits.

Fig 5 shows the distribution of data in this study marked by small black circles that are spread out all the circles are filled with black, and the dots are distributed equally on both sides; this proves that the data collected in this study does not contain elements of bias. However, in systematic reviews, publication bias and related biases might lead to unduly hopeful outcomes. As stated by [39] the funnel plot, which is commonly used to detect such biases, has yet to be empirically evaluated as a visual tool. Researchers employed numerous approaches to assess publication bias, including The Egger Test and the Fail-Safe N. Table 7 displays the Egger test findings.

**Table 7:** Egger test and Fail-Safe N.

Evaluation of Publication Bias		
Test Name	value	p
Fail-Safe N	37588.000	< .001
Begg and Mazumdar Rank Correlation	-0.017	0.900
Egger’s Regression	-0.426	0.670
Trim and Fill Number of Studies	7.000	.
Note. The Rosenthal Approach to Fail-safe N Calculation		

Source: Author’s data processing, 2024

**Fig 6:**
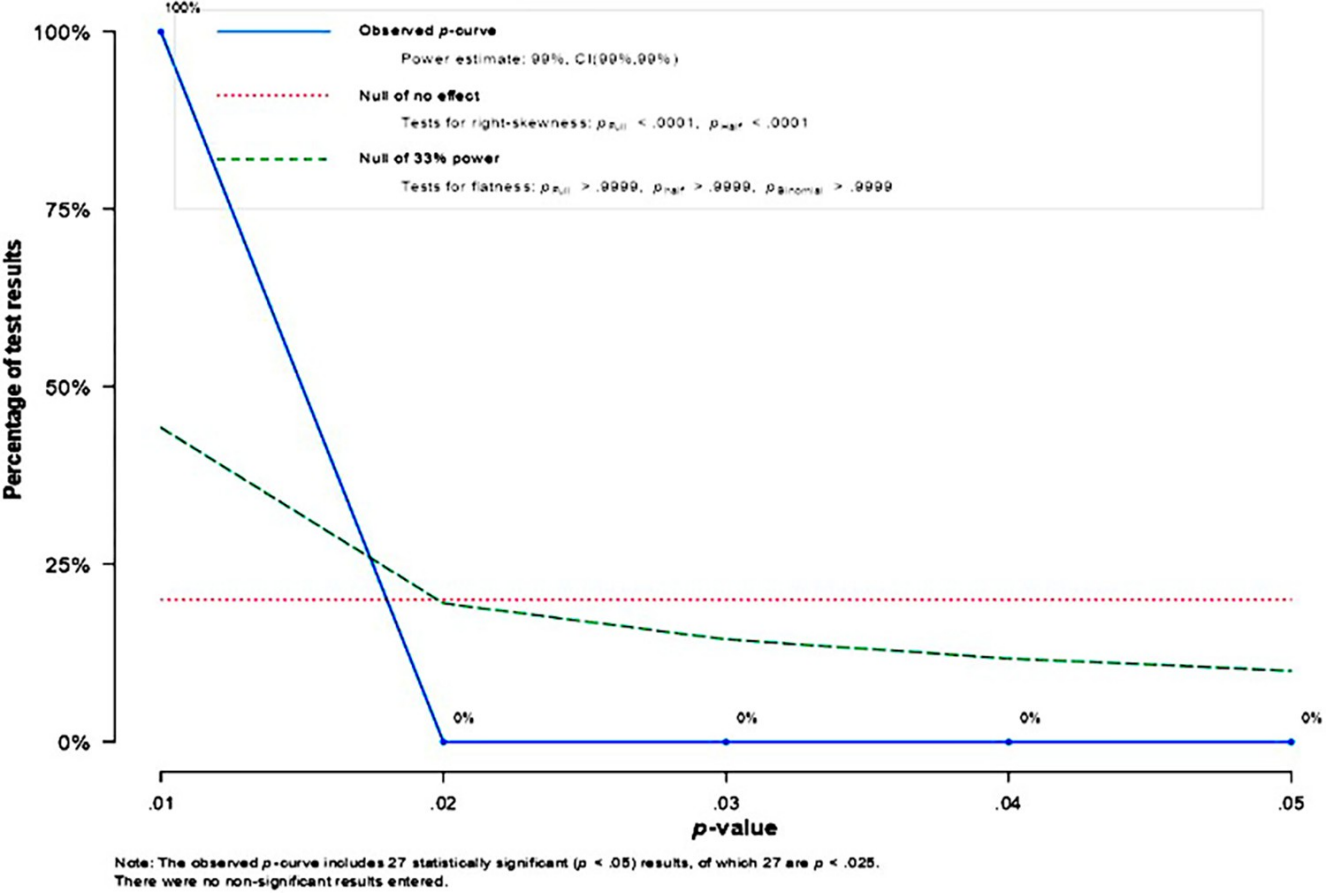
p curve plot. Source: Author’s data processing, 2024.

The Egger test results in Table 7 show a p-value > 0.05 (p = 0.670 > 0.05), indicating that the funnel plot is symmetrical, although the distribution of the plot is not very regular. It can be concluded that there is no issue of publication bias in this meta-analysis study. Publication bias can also be analyzed using fail-safe N values. The fail-safe N value result for the 27 studies analyzed was 37588. This value indicates that 37588 studies had publication bias issues or were not conducted systematically. 37588 studies may not have been reported or published. At the same time, the value of Safe N is greater than the value of 5K + 10 = 5(27) + 10 = 145. Therefore, the foolproof N test concluded that there was no issue of publication bias in this meta-analysis study. Generally, the findings of meta-analyses can be scientifically justified on the basis of publication bias tests. P-curve analysis has been proposed to detect P hacking and publication bias in meta-analyses. The P-curve assumes that publication bias occurs not only because researchers do not publish non-significant results, but also because analysts "play" with their data ("P-hacking"; e.g. selectively removing outliers, choosing different results, different variables were controlled) until non-significant findings became significant (i.e. p < 0.05) [40]. In addition, in order to clarify that this study is not biased, a p-curve was used in this study to avoid the influence of bias. Fig 6 explains the p-curve analysis, including the interpretation of the evidence, the number of studies presented/significance/p<0.025, the power estimate and 95% confidence interval, and two lines indicating whether the strength of the evidence for the results is appropriate.

## Discussion

The analysis results revealed heterogeneity in the effect sizes of the 26 studies assessed based on the heterogeneity test. Turn off heterogeneous conditions based on the following criteria: p-value 0.001; Q = 1171.391; 2 or > 0; I2 (%) = 97.55, near to 100. If heterogeneity is observed in the heterogeneity test results, it signifies that the estimated study criteria assessed indicate a substantial difference, and so the pooled/pooled ES can be interpreted. This heterogeneity test also indicated that this study may move forward with effect size analysis. This is compatible with Müller’s assertion in Ellinor et al. [41] pointed out that before drawing conclusions based on fixed effects patterns, meta-analytic research must first establish the amount of heterogeneity. According to the meta-analysis of 27 researches, TOE has a considerable influence on the performance of SMEs, particularly in the agriculture sector, as evidenced by a p value of 0.01. This is supported by the premise that TOE aims to improve quality and identify the use of smart agriculture in the context of technology, organization, and environment in accordance with consumer expectations of impact, efficiency, and effectiveness, as well as business owners’ experience. Based on the results of data processing, it shows that the adoption of TOE has a positive and significant impact on (a) overall SME agricultural performance, (b) technological performance, (c) organizational performance, and (d) environmental performance, hence H.1 is accepted and H.2 is rejected.

The results of this study are in line with prior research findings that show a direct positive relationship between TOE constructs and SME agriculture performance with variables of technological factors, organizational and environmental factors, and SME performance, and emphasize that the relationship between the evaluation of theoretical and practical contributions to agribusiness and the adoption of TOE is positive [42–47]. SMEs in the agriculture sector need to properly plan and manage their presence on social media in order to utilize its full potential that is targeted and relevant to their target market, in line with Giampietri & Trestini [48] finding stated adopting technology is positively influenced by customers’ readiness to use this technology and perceived ease of use through various forms of social media [49]. Relative advantage, compatibility, security concerns, cost savings; technology readiness, top management support, competitive pressure, and regulatory support all have a significant impact on the adoption of cloud computing-based technology management for SMEs [50, 51]. This is the basis for SMEs agriculture to continue to be able to increase good production results to maintain the stability of food products in the future with technology. However, in the process of technology adoption is certainly not an easy thing, there are several constraining factors in adopting TOE such as technology costs and perceived risk and relative advantage [52], complexity, top management support, and competitive pressure factors [53–55], education (information) about technology, greater access to credit, and incentives [56]. Farmers in rural areas often face limited access to communications infrastructure and internet networks, hindering their ability to adopt technology. Some agricultural technologies may require high initial costs, including purchasing hardware and software, care and maintenance costs, and a lack of resources. Farmers who do not fully understand the benefits and how technology works may be reluctant to adopt it, and government regulations or policies may inhibit the use of certain technologies in the agricultural sector. This proves that empirically, this research found several challenges faced by countries in the developing category in adopting technology to optimize agricultural output. To create an excellent technological environment, it starts from within the company. The company environment plays a vital role in forming the relationship between technical, organizational, environmental, and social factors and the operational performance of SMEs [26]. This avoids internal risks related to organizational dimensions and external threats related to environmental dimensions [57], to enhance technology compatibility, organizational financial costs, and changes in the digital environment influence the adoption of smart agriculture [58].

Growing of globalization and rapid digitalization in numerous industries have resulted in rising worldwide rivalry; in this situation, social media plays an important role in the adoption of technology in various sectors, including agriculture. This study’s findings also imply that the participatory aspect of social media allows for two-way dialogue with stakeholders. Interests, thereby encouraging SMEs to adopt it in line with research Nguyen et al. [59] that shows of the three factors: technological, organizational, and environmental context are the drivers of online marketing for SMEs. Research by Qalati et al. [60] stated the significant influence of the factors relative advantage, cost-effectiveness, compatibility, interactivity (technology), entrepreneurial orientation (organization), and customer pressure (environment), and insignificant influence of the determinants of top management support (organization) and competitive pressure (environment) on social media adoption. Socialization, education, product promotion, discussion and collaboration, performance monitoring, fundraising, and investment, can be a reference for developing countries in overcoming problems in TOE adoption [61].

Furthermore, several advantages of implementing TOE in agricultural SMEs are: *first*, TOE can help farmers increase their agricultural productivity through improvements in soil management, irrigation, fertilization, and monitoring agricultural conditions [62, 63]; *second*, it optimizes better supply chain management [64]; information and communication systems can assist in agricultural supply chain management, enabling product tracking from farmer to consumer [65]. This improves transparency, food safety, and resource tracking. *Third*, for Better food safety, block chain can be used to trace the source of origin of agricultural products [66], when a disease outbreak or food safety problem occurs, block chain enables rapid identification of the root of the problem [55]. This helps reduce the risk of spreading disease or contaminated products to the market. *Fourth*, the intelligent use of technology in agriculture can help reduce the negative impact of agriculture on the environment. This includes reducing pesticide, water, and energy use [67].

In addition, Opasvitayarux et al. [68] explained technology adoption in the agricultural sector (TOE) is influenced by several factors or antecedents that can influence the level of adoption and use of the technology. Some antecedents that influence TOE adoption in the agricultural sector include: *first*, technological infrastructure, the cost of hardware and software, as well as their availability, influences farmers’ ability and willingness to adopt a particular technology similar with prior research [51]. *Second* is education and skills and ability to handle change, implementing toe often requires changes in the way farmers work. They must be able to handle these changes well and be willing to change their farming practices [55]. *Third*, trust in technology, farmers’ trust in technology is an important factor. If farmers do not believe the technology will provide benefits or if they are concerned about privacy or security issues, they may be reluctant to adopt the technology [53]. *Fourth*, leadership and government support; align with research by Qalati et al. [42] support from government, agricultural organizations, and other stakeholders can help facilitate TOE adoption, strong leadership and incentive or financial assistance programs can stimulate technology adoption [54]. *Fifth*, contextual factors such as climate, environment, and soil conditions also influence the type of technology that is most appropriate to implement on a particular region or farm [62]. *Sixth* is commitment to sustainability: farmers who are committed to sustainable farming practices are more likely to adopt technologies that support those goals, such as efficient water use or reduced pesticide use [43]. *Seventh* is how keeping a good relationship with markets, TOE implementation can also be influenced by farmers’ relationships with markets and customers. If farmers see consumer demand for technology-enabled agricultural products, they may be more inclined to adopt the technology [58]. Data security and privacy: in adoption of TOE about data security and privacy are also factors to consider. *Eight*, Farmers should feel confident that their data will be managed securely and in accordance with the law [57].

## Conclusion and recommendation

From the research and discussion above, in order to face the challenges of modern agriculture, TOE adoption is a must for SMEs in the agricultural sector. This not only allows them to compete in increasingly complex markets, but also contributes to global food security and environmental sustainability. Therefore, support and investment in the adoption of agricultural technology is very important for the development of SMEs in the agricultural sector. The synergy between technology adoption by agricultural SMEs and Industry 4.0 can increase connectivity and automation in the agricultural sector. This enables smarter, adaptive and environmentally friendly agricultural production. However, it is important to remember that adopting TOE to realize the smart agriculture concept has its own challenges and risks, such as resource management (technology), good organizational management (organization), and internal and external organizational environments (environments), including intense competition.—these factors are understood and managed well, TOE adoption in the agricultural sector can be more successful and have a positive impact on farmers’ productivity, sustainability and competitiveness. To overcome this challenge, it is very important and regularly emphasized for governments, non-governmental organizations and educational institutions to provide support to agricultural SMEs in the form of training, education, access to capital and technical support. Apart from that, promotion and outreach regarding the benefits of agricultural technology are also very important so that farmers can better understand and adopt this technology.

## Research implication

Over the past few years, there has been an ongoing debate regarding the impact of TOE adoption on the agricultural sector. However, research in this area is still relatively small because the majority of SMEs operating in developing countries avoid adopting TOE due to a lack of weak resources both technologically, organizationally, and in the internal and external environment [30]. This research provides a comprehensive picture of the determining factors that influence TOE adoption and its antecedents, thereby enabling decision-makers, especially top management, to understand the importance of social media adoption and its use in the agricultural sector to realize smart agriculture. Furthermore, this paper broadens awareness of how effective and suitable technology use might boost performance. According to research findings, social media usage has a significant impact on SME performance in terms of strengthening connections and communication with stakeholders, lowering marketing expenditures, and increasing consumer loyalty and retention. Furthermore, TOE adoption improves access to information about competitors and customers. The identified influencing factors provide practitioners and decision-makers with a clearer understanding, allowing them to focus on factors that have a positive and significant impact on TOE adoption, allowing them to be more independent in the context of building business concepts that have an impact, which is self-sufficient in order to adapt to the agricultural technology age 4.0. As a result, policymakers must encourage entrepreneurial activities in SMEs. In conducting research on the challenges of TOE adoption in the agricultural sector, it is important to integrate a multidisciplinary approach, including economics, social science, technology and environmental science. The results of this research can provide deeper insights into how to increase TOE adoption and support the sustainable development of the agricultural sector.

## Supporting information

S1 Checklist PRISMA 2020 for abstracts checklist: A meta-analysis of the impact of TOE adoption on smart agriculture SMEs performance. (PDF)

S2 Checklist PRISMA 2020 checklist: A meta-analysis of the impact of TOE adoption on smart agriculture SMEs performance. (PDF)

S1 File (PDF)

S1 Data (XLSX)

S1 Fig Descriptive figure analysis. Source: Author’s data processing, 2024.
(JPG)
