# Supplementary material for: A meta-analysis of the impact of TOE adoption on smart agriculture SMEs performance
Source: PLoS One. 2025 Feb 3;20(2):e0310105. doi: 10.1371/journal.pone.0310105 (PMC11790137; doi:10.1371/journal.pone.0310105)
Supplement: S1 File — (PDF) [file pone.0310105.s003.pdf]

TY - JOUR
T1 - Determinants of ICT Adoption Among Small Scale Agribusiness Enterprises In Somalia
A1 - Abdullahi, Husein Osman
A1 - Hassan, Abdikarim Abi
A1 - Mahmud, Murni
A1 - Farah Ali, Abdifatah
Y1 - 2021/02//
JF - International Journal of Engineering Trends and Technology
VL - 69
IS - 2
SP - 68
EP - 76
DO - 10.14445/22315381/IJETT-V69I2P210
UR - https://ijettjournal.org/archive/ijett-v69i2p210
N2 - The use of Information and Communication Technology (ICT) can advance the Agricultural Business sector, particularly in a country seeking opportunities to explore the sector. There is evidence that ICT has made significant contributions to agribusiness because it allows enterprises to manage their operations, and it has major impacts on the business. However, the critical factors that motivate the adoption of new innovative technology by agribusiness enterprises are underexplored. The literature has indicated ICT adoption among small-scale agribusiness enterprises in Somalia is not fully understood. Nevertheless, this study addresses this gap by investigating the adoption of ICT among small-scale agribusiness enterprises in Somalia. The paper reports the use of the Technology, Organization, Environment (TOE) framework. An online survey has been conducted with random sampling for data collection, with 107 respondents. The respondents are from agribusiness staff and farmers from various agricultural companies in Somalia. After quantitative data analysis, the results indicated that relative advantage, complexity, top management support, and competitive pressure factors are significant contributors to ICT adoption in Somalian agribusiness enterprises, while ICT costs and vendor support are not significantly related to the adoption of ICT in agricultural business. This study concludes that ICT adoption in Somalia is inspired by insight and motivation rather than financial and external support. Understanding these factors leads to a better understanding of ICT adoption in Somalia. Additionally, it enriches the literature about the agriculture business on the African continent.
ER -

TY - JOUR
T1 - Determinants Impacting the Assimilation Stages of E-commerce in SMEs: A Modified TOE Framework
A1 - Ahmad, Mansoor
A1 - Siraj, Sadaf
Y1 - 2023///
KW - Assimilation
KW - E-commerce
KW - SMEs
KW - TOE Framework
KW - adoption
JF - International Journal of Electronic Commerce Studies
VL - 14
IS - 2
SP - 101

EP - 126
DO - 10.7903/ijecs.2220
N2 - This article aims to examine the factors affecting e-commerce assimilation in Indian SMEs. It proposes a research model based on the technology, organization, and Environment (TOE) framework that includes technological, organizational, environmental, and individual determinants of e-commerce adoption and assimilation. Survey data from 384 SMEs in North India reveals that technological, organizational, environmental, and individual (TOEI) determinants explain e-commerce adaptation. The results show that although Indian SMEs are adopting e-commerce, they cannot reach the final integration stage. Although all factors significantly influence the e-commerce assimilation in SMEs, technological factors have emerged as the most influential predictors of e-commerce adoption among the other elements, viz. The technological context directly affects e-commerce adaptation through organizational, environmental, and individual compatibility. In contrast, technological cost and perceived risk are the most crucial factors inversely impacting e-commerce adaptation. Therefore, managers, policymakers, and government should focus on developing technical infrastructure and providing technical support to SMEs to promote e-commerce integration. It will help them to take advantage of the opportunities that e-commerce offers.
ER -

TY - JOUR
T1 - … and Environment (TOE) Framework Toward Supply Chain Management System Based on Cloud Computing Technology for Small and Medium Enterprises
A1 - Amini, Mahyar
A1 - Javid, Negar Jahanbakhsh
Y1 - 2023///
JF - … for Small and Medium Enterprises …
VL - 11
IS - 8
SP - 1217
EP - 1234
UR -
https://papers.ssrn.com/sol3/papers.cfm?abstract_id=4340207%0Ahttps://www.researchgate.net/profile/Mahyar-Amini/publication/368282581_A_Multi-Perspective_Framework_Established_on_Diffusion_of_Innovation_DOI_Theory_and_Technology_Organization_and_Environme
N2 - … a sample of 22 SMEs that all of these SMEs as a customer’s … technology department from those SMEs are selected to fill the … of supply chain management based on CC for SMEs. …
ER -

TY - JOUR
T1 - Determinants of technology adoption by micro and small enterprises (MSEs) in Awi zone, Northwest Ethiopia
A1 - Andaregie, Adino
A1 - Astatkie, Tessema
Y1 - 2022///
PB - Taylor & Francis
JF - African Journal of Science, Technology, Innovation and Development
VL - 14
IS - 4

SP - 997
EP - 1006
DO - 10.1080/20421338.2021.1923385
N2 - Adoption of technology can enhance the development of micro and small enterprises (MSEs). But in Ethiopia, there is a very low adoption of broadband connections, mobile phones, computers, printers, scanners, copiers, and other technologies by MSEs. The main objective of this study was to identify the determinants of technology adoption by MSEs in Northwest Ethiopia. Cross-sectional data were collected from 327 MSEs selected using the stratified random sampling method and analyzed using the Heckman two-stage model. The first stage probit model estimation results showed that sex, educational level, source of start-up capital, size of the enterprise, and whether the owner of the enterprise has had technology related trainings were significant factors determining technology adoption decisions of MSEs. The second stage estimation results showed that sex, education level, experience, age, family size of the owner, and access to credit significantly influence the degree of technology adoption. The findings indicate that MSEs need more education (information) on technology, greater access to credit, and incentives provided by the government of Ethiopia and other agencies in order to increase their adoption of technology.
ER -

TY - JOUR
T1 - The Role of Technology in Predicting Business Analytics Adoption in SMEs
A1 - Atan, Mislina
A1 - Mahmood, Rosli
Y1 - 2023/02//
KW - Business analytics adoption
KW - SMEs
KW - compatibility
KW - relative advantage
JF - Journal of Information and Knowledge Management
VL - 22
IS - 1
SN - 0219649222500
DO - 10.1142/S0219649222500708
UR - https://www.worldscientific.com/doi/10.1142/S0219649222500708
N2 - Research shows that data-driven decision making using business analytics can create competitive advantages for organisations. However, this can only happen if the organisations successfully accept and use the business analytics effectively. Many studies reported business analytics implementation in large organisations, and fewer studies focus on Small and Medium Enterprises (SMEs). Furthermore, SMEs are scoring lower scores in technology absorption. Therefore, it is essential to examine the business analytics adoption among SMEs. Previous research has reported that relative advantage and compatibility were the most highlighted factors under the technology dimension in adopting innovative technologies. However, the literature reported inconsistent findings on the significance of relative advantage and compatibility in adopting various technologies. Therefore, this research conducted a quantitative survey-based study to examine the significance of relative advantage and compatibility in predicting business analytics adoption among SMEs. The sample was selected using systematic random sampling from a Malaysian national entrepreneurs database. There were 241 SMEs that responded to the online survey sent by email. The analysis using the partial least squares structural equation modelling (PLS-SEM) informed that relative advantage was significantly related to business analytics adoption; however, compatibility did not influence the business analytics adoption by SMEs in Malaysia.

This finding shows that the better the relative advantage of business analytics SMEs know, the higher the possibility of adoption. In addition, less compatibility of the SMEs in Malaysia hindered the business analytics adoption. This study contributes to the theoretical aspect, which statistically informed the finding out of inconsistent gaps in technology adoption. Furthermore, this study also contributes to the practical aspect, in which managers, owners, vendors, and policy-makers can use these findings to spur and facilitate business analytics adoption among SMEs in developing countries.
ER -

TY - JOUR
T1 - Integrated technology-organization-environment (T-O-E) taxonomies for technology adoption
A1 - Awa, Hart O.
A1 - Ojiabo, Ojiabo Ukoha
A1 - Orokor, Longlife E.
Y1 - 2017/10//
KW - Adoption
KW - T-O-E taxonomies
KW - Technology
JF - Journal of Enterprise Information Management
VL - 30
IS - 6
SP - 893
EP - 921
DO - 10.1108/JEIM-03-2016-0079
UR - https://www.emerald.com/insight/content/doi/10.1108/JEIM-03-2016-0079/full/html
L1 - file:///C:/Users/user/AppData/Local/Mendeley Ltd./Mendeley Desktop/Downloaded/Awa, Ojiabo, Orokor - 2017 - Integrated technology-organization-environment (T-O-E) taxonomies for technology adoption.pdf
N2 - The relationship between adoption and the factors within the contexts of technology, organization, environment and task were statistically supported though some had negative coefficients. For individual context, social factor had a statistically significant negative coefficient but hedonistic drive was not statistically supported.
ER -

TY - JOUR
T1 - Understanding and predicting the determinants of blockchain technology adoption and SMEs' performance
A1 - Bag, Surajit
A1 - Rahman, Muhammad Sabbir
A1 - Gupta, Shivam
A1 - Wood, Lincoln C.
Y1 - 2023/12//
KW - Blockchain
KW - Emerging economies
KW - Financial performance
KW - Market performance
KW - Small- and medium-sized enterprises (SMEs)
JF - International Journal of Logistics Management
VL - 34

IS - 6
SP - 1781
EP - 1807
DO - 10.1108/IJLM-01-2022-0017
UR - https://www.emerald.com/insight/content/doi/10.1108/IJLM-01-2022-0017/full/html
N2 - Purpose: The success of SMEs' financial and market performance (MAP) depends on the firms' level of blockchain technology adoption (BCA) and identifying the crucial antecedents that influence SMEs' adoption. Therefore, this research attempts to develop an integrated model to understand and predict the determinants of BCA and its effect on SMEs' performance. The purpose of this paper is to address this issue. Design/methodology/approach: The theoretical foundations are the technology–organization –environment (TOE) framework and the resource-based view (RBV) perspective. The authors distributed a survey to SMEs in South Africa and received 311 responses. The covariance-based structural equation modeling (CB-SEM) followed by the artificial neural network (ANN) technique was used for the data analysis. Findings: The SEM results showed that SMEs' relative advantage, compatibility, top management support (TMS), organizational readiness (ORD), competitive pressures (COP), external support, regulations and legislation significantly influence SMEs' BCA. However, complexity negatively impacts SMEs' BCA. The analysis results also revealed that SMEs' BCA significantly influences the financial performance of the firms, followed by MAP. Furthermore, model determinants were input to an ANN modeling. The ANN results showed that TMS is the most critical predictor of SMEs' BCA, followed by ORD, COP, external support, and regulations and legislation. Practical implications: The results provide valuable information for SMEs when maneuvering their adoption strategies in the scope of blockchain technology. Additionally, from the perspective of an emerging market, the study has successfully contributed the TOE framework and the RBV. Originality/value: This study is the first work to explore the determinants of BCA in the context of SMEs from a developing country. This paper is also one pioneer in attempts to develop a causal and predictive statistical model for predicting the determinants of BCA in SMEs' performance.
ER -

TY - JOUR
T1 - Effect Of Technology, Organization, And External Environment On Business Performance Mediated By The Adoption Of Technology 4.0 In SMEs
A1 - Bawono, Hario Tejo
A1 - Winarno, Winarno
A1 - Karyono, Karyono
Y1 - 2022///
JF - Manajerial
VL - 9
IS - 02
SP - 228
EP - 228
DO - 10.30587/jurnalmanajerial.v9i02.3854
N2 - Background – SMEs are very important for a country's economic growth and are recognized as one of the main contributors to economic development, which results in job growth. However, Manding SMEs in Bantul Regency find it difficult to survive in the current economic environment, especially during the current Covid-19 pandemic. This study analyzes the issue of deteriorating performance due to the COVID-19 pandemic in Bantul. Aim – This study aims to re-analyze the relationship between Technology-Organization-Environment (TOE) and SME business performance. Design / methodology / approach – This research is a census study with the number of respondents as many as 30 UKM Leather

Manding in Bantul, Yogyakarta, Indonesia. The method of data collection is done by distributing questionnaires. The analytical tool used in this research is Partial Least Square (PLS). Results and Discussion - This study has found a positive and significant relationship with each hypothesis. The results of hypothesis 1 in this study found that technology factors were positively and significantly related to SME business performance. hypothesis 2 has the greatest value compared to the variables of technological factors and external environmental factors. hypothesis 3 has a relatively small original sample value compared to technological factors and organizational. hypothesis 4 in this study indicate that the direction of the relationship is positive and significant. hypothesis 5 found that organizational factors affect SME business performance mediated by the adoption of technology 4.0. Conclusion - The results of the research that have been carried out show that the Leather Manding SME in Bantul has SME business performance which is influenced by the adoption of technology 4.0. On the other hand, the adoption of technology 4.0 itself is influenced by technological factors, organizational factors, and external environmental factors. Research implication – Provide direction for SME Kulit Manding in Bantul to strengthen and sustain organizational growth by enhancing and collaborating on SME's distinct image, goals, strategies, and core values as well as facilitating consistent relationships within and outside of ICT interactions in order to compete and excel in the leather craft industry. Limitations – Based on the findings of this study, this study has several limitations, the sample in this study was limited to certain areas, namely Manding, Bantul, Special Region of Yogyakarta. Although this study establishes a cognitive model of the TOE and SMEs business performance consistent with the theoretical basis used, however, based on theoretical aspects, the study of technology adoption 4.0 in SMEs still provides development opportunities to test the cognitive model TOE and SMEs business performance.
ER -

TY - JOUR
T1 - Testing the adoption of Blockchain Technology in Supply Chain Management among MSMEs in China
A1 - Deng, Nianqi
A1 - Shi, Yangyan
A1 - Wang, Junbin
A1 - Gaur, Jighyasu
Y1 - 2022/08//
KW - Blockchain technology adoption
KW - MSMEs
KW - Supply chain management
JF - Annals of Operations Research
DO - 10.1007/s10479-022-04856-4
UR - https://link.springer.com/10.1007/s10479-022-04856-4
N2 - Supply chain (SC) digitalization has become a new trend in the development of micro, small, and medium enterprises (MSMEs). Blockchain technology (BCT) is a cutting-edge innovation that many supply chain management (SCM) professionals have already adopted. Although studies on BCT have yielded some findings, they do not provide sufficient discussion regarding BCT adoption in SCs among MSMEs. Moreover, the determinants and effects of BCT adoption on SCM among MSMEs remain unclear. This study aims to bridge these gaps by helping understand individual BCT adoption in the SC domain in Chinese MSMEs. Using the technology-organization-environment (TOE) framework, this study examines the effects of BCT, organizational, and environmental contexts on BCT adoption in MSMEs' SCs. The findings reveal that cost saving, complexity, relative advantage, top management support, SC cooperation, and government support positively affect BCT adoption in SCM. Whereas, compatibility,

technological readiness, financial readiness, and competitive pressure had no significant impact on BCT adoption in SCM among MSMEs in China.
ER -

TY - JOUR
T1 - Analysing farmers' intention to adopt web marketing under a technology-organisation-environment perspective: A case study in Italy
A1 - Giampietri, Elisa
A1 - Trestini, Samuele
Y1 - 2020/05//
JF - Agricultural Economics (Zemědělská ekonomika)
VL - 66
IS - 5
SP - 226
EP - 233
DO - 10.17221/355/2019-AGRICECON
UR - http://agricecon.agriculturejournals.cz/doi/10.17221/355/2019-AGRICECON.html
N2 - This study explores the factors that affect the intention to adopt web marketing (WM) at farm level as an innovation for business purposes. Data were collected from a direct survey among Italian farmers. The paper applies the Technology-Organisation-Environment (TOE) framework. Among the variables considered, the results mainly show that a higher perceived usefulness of WM leads to a greater intention to adopt it from farmers. Similarly, the intention to adopt WM is positively influenced by the customers‘ readiness to use this technology and the perceived ease of use. Moreover, the intention to adopt is lower for the farms showing a greater size. Surprisingly, the perception of cus-tomers’ positive expectation about WM adoption by the farm shows a negative effect, suggesting that external pressures can inhibit farmers’ intention. Findings are valuable to understand how to develop policies to support WM adoption among farmers, that is important to gain access to the market especially for smallholders.
ER -

TY - JOUR
T1 - The adoption stages (Evaluation, Adoption, and Routinisation) of ERP systems with business analytics functionality in the context of farms
A1 - Junior, Caetano Haberli
A1 - Oliveira, Tiago
A1 - Yanaze, Mitsuru
Y1 - 2019/01//
PB - Elsevier
JF - Computers and Electronics in Agriculture
VL - 156
IS - December 2018
SP - 334
EP - 348
DO - 10.1016/j.compag.2018.11.028
UR - https://linkinghub.elsevier.com/retrieve/pii/S0168169918301030
N2 - Agriculture is a complex industry based on science. Agriculture relies on systems of climate-standard analysis: solar energy, heat, moist and systems for the area of field operation patterns: soil chemical composition, plant nutrition, genetic improvement, pest and disease control, harvesting. We believe that the new paradigm to increase efficiency in this segment goes through the adoption of

enterprise resource planning (ERP) systems. Our empirical study is about the use and diffusion of ERP systems in a view of interoperability between different software packages with a view on business analysis functionality when taking a step further in farm management information system (FMIS). We hope this work can bring a theoretical and practical contribution for the agribusiness field and also increase debates about the platforms on cloud computer based on ERP, Enterprise 2.0 and Industry 4.0. The research presented in this study was carried out with 375 farmers in Brazil. The data gathering instrument used for the quantitative research was built based on the result of the qualitative (in-depth) study in combination with three theories: Diffusion of Innovation Theory (DOE), Technology-Organization-Environment Framework (TOE), and Inter-organizational Relations (IOR). The construct information sharing (IS) taken from IOR was applied to perform a moderator role on the measurement of ERP adoption stages. The results indicate the significant drivers for evaluation, adoption, and routinisation. Also, as a result, it was found that Information Sharing influence the relationship between evaluation and adoption positively. Moreover, the theoretical and managerial implications of the research results are also debated in the paper.
ER -

TY - JOUR
T1 - Determinants of Collaborative Robots Innovation Adoption in Small and Medium-Sized Enterprises: An Empirical Study in China
A1 - Liu, Dong
A1 - Cao, Junwei
Y1 - 2022/10//
KW - collaborative robots
KW - diffusion of innovations theory
KW - innovation adoption
KW - small and medium-sized enterprises
KW - technology-organization-environment framework
JF - Applied Sciences
VL - 12
IS - 19
SP - 10085
EP - 10085
DO - 10.3390/app121910085
UR - https://www.mdpi.com/2076-3417/12/19/10085
N2 - With the rapid development of industry 4.0 and the boom of large-scale product customization, the adoption of collaborative robots' innovation becomes a hot topic in research. Previous studies have mainly focused on individuals, but few on enterprises, and in particular, there has been a lack of empirical research on the enterprise level. Based on the combined model of Technology-Organization-Environment Framework (TOE) and Diffusion of Innovations Theory (DOI), this study investigated 373 small and medium-sized enterprises (SMEs) in Guangdong Province, China, to explore the determinants of SMEs' adoption of collaborative robot innovation in technology, organization, and environment. The result shows that the technical factors of relative advantage, compatibility, observability, and trialability have a significant positive correlation with the adoption of collaborative robots, while complexity has a significant negative correlation with the adoption. Among the organizational factors, top management support and organizational readiness have a significant positive correlation with the adoption of collaborative robots. Among the environmental factors, agent support is positively and significantly correlated with adoption. The findings will help practitioners develop appropriate strategies for the adoption of collaborative robot innovation.

ER -

TY - JOUR
T1 - Influence of Digital Accounting System Usage on SMEs Performance: The Moderating Effect of COVID-19
A1 - Lutfi, Abdalwali
A1 - Alkelani, Saleh Nafeth
A1 - Al-Khasawneh, Malak Akif
A1 - Alshira’h, Ahmad Farhan
A1 - Alshirah, Malek Hamed
A1 - Almaiah, Mohammed Amin
A1 - Alrawad, Mahmaod
A1 - Alsyouf, Adi
A1 - Saad, Mohamed
A1 - Ibrahim, Nahla
Y1 - 2022///
KW - COVID-19
KW - DAS performance
KW - DAS usage
KW - SMEs
KW - digital-based accounting system (DAS)
KW - resource-based view
KW - technology-organization-environment framework
JF - Sustainability (Switzerland)
VL - 14
IS - 22
SP - 1
EP - 23
DO - 10.3390/su142215048
N2 - In the literature, studies have evidenced the efforts adopted by firms to develop digital technology with the hope of achieving sustainable decisions and competitive performance. However, studies have yet to provide an extensive explanation of the mechanisms used by firms in their digital technology adoption to impact and enhance value, particularly among small and medium enterprises (SMEs). In this regard, accounting information has served as a fundamental basis for business decision-making and the extensive use of digital technology has paved the way for the efficiency and effectiveness of accounting functions in modifying information relating to such functions. More specifically, a digital accounting system (DAS) enables the reporting and processing of large transaction amounts and generates the data required for analysis. However, despite these advantages, SMEs have been slow in their adoption and usage of DASs. Accordingly, this study drew upon resource-based view theory and the technology-organization-environment framework to propose an integrated model for examining the determinants and impact of using DAS among SMEs. The proposed model encapsulates the use and performance aspect of DAS. The study utilized a self-administered survey questionnaire as the primary data collection instrument. Data from 183 SMEs in Jordan were analyzed using partial least squares-structural equation modeling. The findings reveal that compatibility, organizational readiness, top management support and government support all had significant effects on DAS usage, which, in turn, had a positive and significant effect on DAS performance. With regard to the moderating effects, COVID-19 was found to have a moderating role on the DAS usage–DAS performance relationship. The study findings explain the way firms can enhance their DAS use to obtain optimum performance, thereby contributing to the

literature on the antecedents and effects of using current information technology/information systems. The study recommends that the government of Jordan prepare and carry out a campaign concerning the importance of DASs for SMEs.
ER -

TY - JOUR
T1 - Factors Affecting the Adoption of Online Marketing Channels in SMEs: An Empirical Survey in the North of Vietnam
A1 - Nguyen Phuc Hien
A1 - Nguyen Thi Huong
A1 - Nguyen Thi Xuan Thu
Y1 - 2023/04//
JF - VNU University of Economics and Business
VL - 3
IS - 2
SP - 79
EP - 79
DO - 10.57110/vnujeb.v3i2.162
UR - https://jeb.ueb.edu.vn/index.php/jeb/article/view/162
N2 - This study attempts to investigate the awareness of SMEs in some northern provinces of Vietnam about online marketing, to determine the factors encouraging the SMEs to use online marketing channels, and to measure to what extent they influence the adoption. The quantitative method measures the intensity relationship between determinants in the Technology, Organization, and Environment (TOE) based theoretical model. In combination with it, the qualitative method is applied for the first phase of research exploration. Relying on the research model, a questionnaire is designed to survey 234 SMEs in some northern provinces of Vietnam. Multivariate regression is run with the help of software SPSS 22 to test which factors influence the adoption of online marketing. The result indicates that all three factors of the technological, organizational, and environmental context are drivers of online marketing for SMEs in some northern provinces of Vietnam. However, the organizational factor is the most important one. Based on these important results, the study proposes implications for SMEs to improve their marketing activities.
ER -

TY - JOUR
T1 - Antecedents of IoT adoption in food supply chain quality management: an integrative model
A1 - Opasvitayarux, Pakorn
A1 - Setamanit, Siri-on
A1 - Assarut, Nuttapol
A1 - Visamitanan, Krisana
Y1 - 2022/12//
JF - Journal of International Logistics and Trade
VL - 20
IS - 3
SP - 135
EP - 170
DO - 10.1108/JILT-05-2022-0002
UR - https://www.emerald.com/insight/content/doi/10.1108/JILT-05-2022-0002/full/html

N2 - Purpose – The introduction of quality management Internet of things (QM IoT) can help food supply chain members to enhance real-time visibility, quality, safety and efficiency of products and processes. Current literature indicates three main research gaps, including a lack of studies in QM IoT in the food supply chain, the vagueness of integrative adoption of new technology framework and deficient research covering both adoption attitude and intention in the same model. This study aims to propose an analysis model based on the technological–organizational–environmental (TOE) framework and reinforced by the collaborative structure to capture the importance of the supply chain network. Design/methodology/approach – The partial least square-structural equation modeling (PLS-SEM) was applied to test the impacts of the adoption factors on QM IoT adoption attitude and intention among 197 respondents in food manufacturing in Thailand. Findings – The results indicated that compatibility, trialability, adaptive capacity, innovative capability, executive support, value chain partner pressure, presence of service provider and information sharing significantly impacted the attitude toward QM IoT adoption, while adaptive capability, innovative capability and information sharing directly influenced the QM IoT adoption intention. Furthermore, the attitude toward QM IoT adoption positively impacted the QM IoT adoption intention. Practical implications – This study contributed to academicians by proposing a more solid adoption framework for QM IoT area. In addition, the business practitioners could actively prepare themselves for the QM IoT adoption, whereas the service providers could provide better and suitable service. Originality/value – This research contributes to the building of a more solid framework and indicates significant factors that impact the attitude toward QM IoT adoption and adoption intention.
ER -

TY - JOUR
T1 - A mediated model on the adoption of social media and SMEs’ performance in developing countries
A1 - Qalati, Sikandar Ali
A1 - Yuan, Li Wen
A1 - Khan, Muhammad Aamir Shafique
A1 - Anwar, Farooq
Y1 - 2021/02//
PB - Elsevier Ltd
JF - Technology in Society
VL - 64
IS - July 2020
SP - 101513
EP - 101513
DO - 10.1016/j.techsoc.2020.101513
UR - https://linkinghub.elsevier.com/retrieve/pii/S0160791X20313166
N2 - Purpose: Small and medium-sized enterprises (SMEs) can use social media for communicating information with stakeholders with minimal cost. The ability to access and share information influences the SMEs' performance, but there is little scholarship on the association between the adoption of social media and SMEs' performance. This study aims to investigate the effects of technology-organizational-environmental (TOE) factors on the adoption of social media and SMEs’ performance in developing countries. Design/methodology/approach: This study employed the TOE framework as determinants affecting the adoption of social media and SMEs' performance. The paper used a self-administered closed-ended questionnaire to gather data, from July 2019 to December 2019, from randomly selected respondents from SMEs operating in Pakistan. Partial-least- squares structural equation modeling was used for the path analysis of 423 responses from SMEs' owners, executives, and managers. It also examines the mediating role of social media between TOE characteristics and SMEs’ performance.

Findings: The findings revealed a direct positive relationship between TOE constructs, the adoption of social media, and SMEs' performance. Full mediation was found between technological factors and SMEs performance, and partial mediation was found between organizational and environmental factors and SMEs’ performance. Complementary mediation among the variables was also examined.
Originality/value: This paper has implications for practitioners and researchers interested in investigating social media adoption in SMEs. It builds an empirical, multi-dimensional hypothesized model, including several determinants that may influence the adoption of social media.
ER -

TY - JOUR
T1 - Social Media and SMEs’ Performance in Developing Countries: Effects of Technological-Organizational-Environmental Factors on the Adoption of Social Media
A1 - Qalati, Sikandar Ali
A1 - Ostic, Dragana
A1 - Sulaiman, Mohammad Ali Bait Ali
A1 - Gopang, Aamir Ali
A1 - Khan, Asadullah
Y1 - 2022/04//
JF - SAGE Open
VL - 12
IS - 2
SP - 215824402210945
EP - 215824402210945
SN - 2158244022109
DO - 10.1177/21582440221094594
UR - http://journals.sagepub.com/doi/10.1177/21582440221094594
N2 - Increasing globalization and rapid digitization across industries have led to greater international competition. Furthermore, the emergence of new innovation has created both challenges and opportunities for small and medium-sized enterprises (SMEs). SMEs have recently been attracted to social media applications to reach a larger audience, improve their relationship with potential customers, and retain existing customers. However, the scant focus has been devoted to comprehensively understanding the adoption of social media in the SME context in developing countries. This study investigates the effects of technological-organizational-environmental (TOE) factors on social media adoption and its effect on SME performance. Data was collected by generating online survey link. SmartPLS 3.3 was used for the path analysis of 381 SMEs. The findings revealed a significant effect of relative advantage, cost-effectiveness, compatibility, interactivity (technological), entrepreneurial orientation (organizational), and customer pressure (environmental) factors, and an insignificant effect of top management support (organizational) and competitive pressure (environmental) determinants on social media adoption. The study found a significant influence of social media on SME performance. This paper offers several implications for decision-makers, policy-makers, and scholars interested in social media and its use. It builds an empirical, integrated framework for SMEs in developing countries.
ER -

TY - JOUR
T1 - Open Banking: The Emergence of New Digital Business Models
A1 - Ramdani, Boumediene
A1 - Rothwell, Ben

A1 - Boukrami, Elias
Y1 - 2020/08//
JF - International Journal of Innovation and Technology Management
VL - 17
IS - 05
DO - 10.1142/S0219877020500339
UR - https://www.worldscientific.com/doi/10.1142/S0219877020500339
N2 - Open banking has recently been advanced as a measure to foster competition and innovation in the retail banking sector. Since its introduction in the UK, a number of banks have created new digital business models (BMs) that offer individuals and businesses access to more personalized financial services. Yet, it is still unclear what new entrants (smaller and newer banks) have done to potentially disrupt incumbents (larger and well-established banks). To shed light on the innovations in BMs that have been initiated by digital banks to move away from traditional retail banking BM, seven digital BMs operating in the UK financial sector were examined using the BM innovation analysis framework. Our findings suggest that innovation in the new digital BMs has been achieved by building on the existing retail banking activities, developing new digitally enabled activities, and leveraging open innovation activities. Implications of our findings for researchers, managers and policy makers will be outlined.
ER -

TY - JOUR
T1 - Digital Technology Adoption in SMEs: What Technological, Environmental and Organizational Factors Influence SMEs' ICT Adoption in Emerging Countries?
A1 - Shahadat, M. M.Hussain
A1 - Nekmahmud, Md
A1 - Ebrahimi, Pejman
A1 - Fekete-Farkas, Maria
Y1 - 2023/01//
KW - Bangladesh
KW - DOI
KW - Digital technology
KW - ICT
KW - SMEs
KW - TOE
KW - developing countries
KW - environmental factor
KW - organizational factor
KW - structural equation modelling
KW - technological factor
JF - Global Business Review
IS - February
SP - 097215092211371
EP - 097215092211371
DO - 10.1177/09721509221137199
UR - http://journals.sagepub.com/doi/10.1177/09721509221137199
N2 - The research aims to explore the technological, environmental and organizational factors that influence digital technology adoption in the small and medium enterprises (SMEs) sector in developing countries. A conceptual framework is proposed that combines the technology-organization-environment (TOE) framework and diffusion of innovation (DOI) theory to explore the determinants of digital

technology, for example, information and communication technology (ICT) applicability in SMEs. In addition, this study identifies the major barriers to implementing ICT in the SMEs sector. A purposive 535 sample was accumulated from higher- and middle-level managers of SMEs firms through a structured questionnaire. Data and hypotheses were analyzed using partial least squares-structural equation modelling. Results show that relative advantage, complexity, observability, perceived cost, top management support, innovativeness of top management, competitive pressure and government support have significant determinants of ICT adoption in SMEs. Besides, compatibility, perceived trialability and organization readiness have not significantly influenced to use of ICT in SMEs. This research has crucial implications for practising managers, policymakers and SMEs business owners. To the best of the authors' knowledge, this study is perhaps one of the first empirical surveys that widely provides a theoretical framework regarding ICT adoption in SMEs in Bangladesh.
ER -

TY - JOUR
T1 - Exploring Risks in the Adoption of Business Intelligence in SMEs Using the TOE Framework
A1 - Stjepić, Ana-Marija
A1 - Pejić Bach, Mirjana
A1 - Bosilj Vukšić, Vesna
Y1 - 2021///
JF - Journal of Risk and Financial Management
VL - 14
IS - 2
SP - 58
EP - 58
DO - 10.3390/jrfm14020058
N2 - The business success of small- and medium-sized enterprises (SMEs) increasingly relies on the adoption of various technological innovations. For today's unpredictable business operations, business intelligence systems (BISs) represent one of the most prominent tools with a significant impact on business performance. However, different internal and external risks may influence BIS adoption. The goal of this paper is to investigate the risks that impact BIS adoption in SMEs, using the Technology, Organization, and Environment (TOE) framework. For that purpose, we develop the logistic regression model, using data collected by a questionnaire survey using a sample of 100 Croatian SMEs. The results indicate the applicability of the TOE theoretical framework for examining BIS adoption in SMEs. Given the results obtained, the sampled SMEs should take into account the internal risks related to the organizational dimension and external risks related to the environmental dimension. Our research did not reveal the significant impact of technological risks that encompass characteristics of considered technological innovation related to the technology dimension.
ER -

TY - JOUR
T1 - Resistance to Agricultural Commercialization with Lack of Marketing Digital Adoption in Indonesia's Dieng Plateau
A1 - Sugandini, Dyah
A1 - Effendi, Mohamad Irhas
A1 - Sugiarto, Bambang
A1 - Kundarto, Muhammad
A1 - Kawuryan, Siwi Hardiastuti Endang
Y1 - 2023///

KW - commercialization resistance
KW - digital marketing
KW - innovation characteristics
KW - production factors
JF - International Journal of Sustainable Development and Planning
VL - 18
IS - 6
SP - 1715
EP - 1724
DO - 10.18280/ijsdp.180607
N2 - Dieng Plateau is one of the largest vegetable-producing areas in Indonesia, and most of the inhabitants work as farmers. While shifting to commercial agriculture with marketing digital skill can improve farmers' lives, it faces obstacles that cause resistance. The barriers that arise from the commercialization of agriculture in Indonesia are that the scale of agricultural business is generally relatively small, capital is limited, and the use of technology is still simple. Agriculture in the Dieng plateau is seasonal and relies heavily on family labor, with limited access to credit, technology, and markets. Wholesalers and the lack of supply of quality seeds for farmers mainly dominate the market for agricultural products. So, this research explains the obstacles that cause farmers to resist commercialization. The observed barriers included five factors: barriers from factors of production and innovation, such as difficulty adopting digital marketing, lack of relative advantages, lack of compatibility, and complexity. This study tests a constraint model of commercial farming using five factors. The data was collected from 280 farmers who own their land and are not farm laborers. The sampling technique used was purposive sampling with the criteria of individuals having a livelihood as farmers, aged more than 18 years, and owning their land. Data collection uses a questionnaire that has a five-point Likert scale—data analysis technique using PLS-SEM. The results support the hypothesis, suggesting a robust barrier to the commercialization model.
ER -

TY - JOUR
T1 - TOE factors and value chain effects of e-business adoption on SMEs
A1 - Thaha, Abdurrahman Rahim
A1 - Maulina, Erna
A1 - Muftiadi, R. Anang
A1 - Alexandri, Mohammad Benny
Y1 - 2022///
JF - Uncertain Supply Chain Management
VL - 10
IS - 3
SP - 1029
EP - 1036
DO - 10.5267/j.uscm.2022.2.009
UR - http://www.growingscience.com/uscm/Vol10/uscm_2022_15.pdf
N2 - The purpose of this study is to determine the effect of the Technology-Organization-Environment (TOE) on e-business adoption and the impact of e-business adoption on the value chain of Small and Medium Enterprises (SMEs). The study uses a quantitative approach where the respondents are 389 SMEs that use e-business in Indonesia. Partial Least Square - Structural Equation Modeling analysis is used in this study to analyze data and test hypotheses. The study results describe that technology, organization, and environment significantly influence the e-business adoption of SMEs. Furthermore, e-

business adoption has a substantial impact on the value chain of SMEs. The study provides an overview of SMEs in Indonesia to determine the factors used as references in e-business adoption and how good e-business adoption will add value to businesses, especially in the value chain.
ER -

TY - JOUR
T1 - Factors Influencing Social Media Adoption Among Smes During Covid-19 Crisis
A1 - Trawnih, Ali
A1 - Yaseen, Husam
A1 - Al-Adwan, Ahmad Samed
A1 - Alsoud, Anas Ratib
A1 - Jaber, Omar Abdel
Y1 - 2021///
KW - Jordan
KW - SMEs
KW - Social media
KW - TAM
KW - TOE
JF - Journal of Management Information and Decision Sciences
VL - 24
IS - 6
SP - 1
EP - 18
L1 - file:///C:/Users/user/AppData/Local/Mendeley Ltd./Mendeley Desktop/Downloaded/Trawnih et al. - 2021 - Factors Influencing Social Media Adoption Among Smes During Covid-19 Crisis.pdf
N2 - Social media is considered a dominant platform for ensuring business success and survival, especially in the case of small and medium enterprises businesses (SMEs). It offers many benefits for businesses in terms of enhancing customer relations, increasing profit, reducing cost, and allowing flexibility. During the Covid-19 pandemic crisis, social media has become essential for businesses. The focus has shifted to conducting businesses' daily activities remotely. Despite its importance, this research aims to explore factors that affect social media implementation on the part of SMEs in Jordan. The Technological, Environmental, and Organizational (TOE) and the Technology Acceptance Model (TAM) has been employed. These theories provide useful insights and explanations with regard to the internal and external contexts of social media adoption. Data was collected from 250 SMEs in the city of Irbid, north Jordan. The results reveal that all factors significantly affect social media adoption on the part of SMEs. Among these factors, environmental context was the most significant predictor of social media adoption during the Covid-19 pandemic crisis; this finding could provide a good basis for SME decision-makers and practitioners regarding assessing the factors that influence social media implementation on the part of SMEs.
ER -

TY - JOUR
T1 - Factors affecting adoption of smart farms: The case of Korea
A1 - Yoon, Cheolho
A1 - Lim, Dongsup
A1 - Park, Changhee
Y1 - 2020/07//
PB - Elsevier Ltd

JF - Computers in Human Behavior
VL - 108
IS - May 2019
SP - 106309
EP - 106309
DO - 10.1016/j.chb.2020.106309
UR - https://linkinghub.elsevier.com/retrieve/pii/S0747563220300637
N2 - The smart farm, a future-oriented farm operation that integrates information and communications technologies, is an emerging trend in agriculture. This study investigates the factors affecting the adoption of the smart farm in Korea and analyzes them empirically. The research model is based on Rogers' innovation diffusion theory and existing models of adoption of information technology in organizations. The model proposes that adoption of innovative technology is influenced by relative advantages, complexity, and compatibility of the technology, the innovativeness and IT knowledge characteristics of the CEOs, financial costs, human resource vulnerability and lack of skills, competitive pressure, government support and the change to the digital environment. These factors were categorized according to TOE framework, investigated, and empirically tested using survey data to determine their influence on the adoption of smart farms. The results showed that the compatibility of technology, financial costs for the organization, and the digital environment change influence the adoption of smart farms. This study suggests practical implications for the adoption of smart farm technology based on the results.
ER -

TY - JOUR
T1 - Analysis of E-Commerce Adoption by SMEs Using the Technology - Organization - Environment (TOE) Model: A Case Study in Karawang, Indonesia
A1 - Setiyani, Lila
A1 - Yeny Rostiani
Y1 - 2021/07//
JF - International Journal of Science, Technology & Management
VL - 2
IS - 4
SP - 1113
EP - 1132
DO - 10.46729/ijstm.v2i4.246
UR - https://ijstm.inarah.co.id/index.php/ijstm/article/view/246
N2 - E-commerce as a medium for online transactions by business actors can increase the productivity of SMEs. This study aims to analyze the adoption of e-commerce in SMEs in Karawang Regency, Indonesia. The technology-organization-environment (TOE) framework was chosen as a variable for measuring e-commerce adoption. Data collection was carried out through a questionnaire survey which was distributed to SMEs in Karawang Regency, and obtained 301 respondents. The results of data collection by using Smart PLS with the results of the technological aspects have no significant effect on the intention to adopt e-commerce, while the organizational and environmental aspects have a significant effect on the intention to adopt e-commerce. All technology indicators, namely compatability, perceived usefulness, complexity, security concern and relative advantage, are proven to have a significant effect on technology in the intention to adopt e-commerce. Organizational indicators, namely cost, organization readiness, organization culture, organization size and top management support, are proven to have a significant effect on organizations in their intention to adopt e-commerce. Meanwhile, environmental indicators, namely government support, competitive pressure,

environmental uncertainty and vendor quality, have a significant effect on the environment in the intention to adopt e-commerce. The data that has been generated can be used by the MSMEs management agency in Karawang Regency to formulate strategies for increasing the productivity of SMEs.
ER -

TY - JOUR
T1 - Empirical investigation of extended TOE model on Corporate Environment Sustainability and dimensions of operating performance of SMEs: A high order PLS-ANN approach
A1 - Dadhich, Manish
A1 - Hiran, Kamal Kant
Y1 - 2022/08//
JF - Journal of Cleaner Production
VL - 363
SP - 132309
EP - 132309
DO - 10.1016/j.jclepro.2022.132309
UR - https://linkinghub.elsevier.com/retrieve/pii/S0959652622019138
ER -

TY - JOUR
T1 - Seizing technological advancement; determinants of blockchain supply chain finance adoption in Ghanaian SMEs
A1 - Asante Boakye, Elijah
A1 - Zhao, Hongjiang
A1 - Coffie, Cephas Paa Kwasi
A1 - Asare-Kyire, Lydia
Y1 - 2023/01//
KW - Blockchain technology
KW - SMEs
KW - TOE
KW - supply chain finance
JF - Technology Analysis & Strategic Management
SP - 1
EP - 17
DO - 10.1080/09537325.2022.2163384
UR - https://www.tandfonline.com/doi/full/10.1080/09537325.2022.2163384
N2 - Blockchain has attracted attention within business circles because it promotes secure and cost-saving transactions between anonymous parties without human mediation. This study examines the Technological Organizational and Environmental (TOE) drivers of blockchain supply chain finance adoption in Ghanaian SMEs. Specifically, this study differs from prior studies employing the United Theory of Acceptance and Use of Technology (UTAUT) and the Technology Acceptance Model (TAM) which ignores the TOE elements. Utilising the Partial Least Squares Structural Equation Modelling method (PLS-SEM) and nonlinear non-compensatory PLS-ANN approach, we provide empirical evidence using data from 214 registered SME members of the Association of Ghana Industries (AGI). Our findings suggest that relative advantage, cost, and complexity significantly influence the adoption of blockchain supply chain finance in SMEs. However, competitive pressure, market dynamics, and SME owner/manager support had no statistically significant influence on the intention of SMEs to adopt

blockchain supply chain finance. This is because of the relatively low adoption rate of blockchain applications in SMEs in the country. Therefore, extensive education on the applications of blockchain technology is required to stimulate diffusion amongst SMEs.

ER -
