# Supplementary figures and images for: A meta-analysis of the impact of TOE adoption on smart agriculture SMEs performance
Source: PLoS One. 2025 Feb 3;20(2):e0310105. doi: 10.1371/journal.pone.0310105 (PMC11790137; doi:10.1371/journal.pone.0310105)

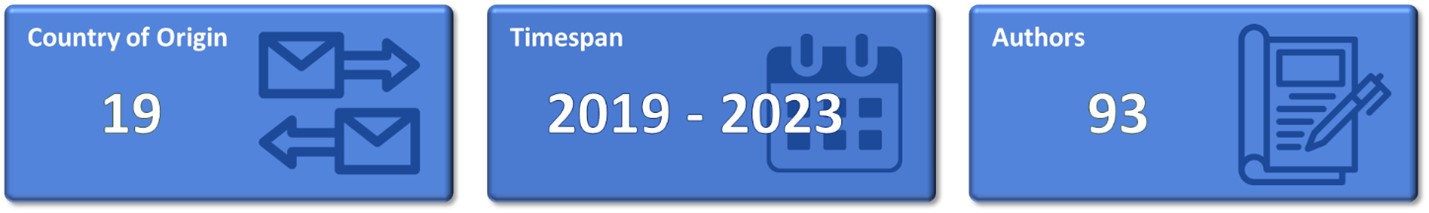

Supplement: S1 Fig — Source: Author’s data processing, 2024. (JPG) [file pone.0310105.s005.jpg]
